# Type 2 gene expression signature in severe asthma associates with more advanced airway remodeling

**DOI:** 10.1002/clt2.70060

**Published:** 2025-06-12

**Authors:** Bogdan Jakiela, Karolina Górka, Iwona Gross‐Sondej, Sławomir Mikrut, Krzysztof Okoń, Piotr Sadowski, Anna Andrychiewicz, Hanna Plutecka, Tomasz Stachura, Grażyna Bochenek, Stanisława Bazan‐Socha, Krzysztof Sładek, Jerzy Soja

**Affiliations:** ^1^ 2nd Department of Internal Medicine Jagiellonian University Medical College Kraków Poland; ^2^ Department of Pulmonology and Allergology University Hospital Kraków Poland; ^3^ Faculty of Mining Surveying and Environmental Engineering AGH University of Science and Technology Kraków Poland; ^4^ Department of Pathology Jagiellonian University Medical College Kraków Poland; ^5^ Department of Endoscopy University Hospital Kraków Poland

**Keywords:** airway remodeling, asthma endotypes, endobronchial ultrasound, severe asthma, type 2 immunity

## Abstract

**Background:**

Asthma is a heterogeneous disease with various inflammatory subtypes, including the type‐2 (T2) endotype associated with airway eosinophilia. Severe asthma is linked to reduced ventilatory function due to airway structural changes. This study compared the extent of airway remodeling in different immunological endotypes of asthma.

**Methods:**

Severe asthma patients (*n* = 30) were stratified based on bronchial expression of T2 (e.g., *CST1*) and T3 (e.g., *IL1*7A) immunity genes as T2‐high, T3‐high, or low‐inflammatory. We analyzed airway wall thickness using endobronchial ultrasound (EBUS), bronchial biopsy morphometry, and mRNA expression of remodeling genes. Bronchial epithelial cell cultures were used to assess cytokine responses.

**Results:**

T2‐high asthma patients showed lower predicted FEV1 (59 vs. 74 % in low‐inflammatory variant, *p* = 0.049) and increased submucosa layer (L2) in EBUS (0.203 vs. 0.189 mm, *p* = 0.018). T2‐high asthma patients also had increased airway smooth muscle (ASM) mass (∼2‐fold, *p* = 0.018) and marginally thicker reticular basement membrane. T3‐high asthma showed only a trend toward thicker L2 (*p* = 0.055). Only patients with an eosinophilic signature in endobronchial biopsy demonstrated increased expression of remodeling genes, including *TGFB1*. A profibrotic profile was also induced in bronchial epithelium stimulated in vitro with IL‐13.

**Conclusion:**

These data suggest that T2‐signature in severe asthma is associated with increased ASM mass and more pronounced airway obstruction. Overexpression of remodeling genes primarily occurred in patients with signs of eosinophilic infiltration in the bronchial mucosa, suggesting that remodeling may progress with uncontrolled airway inflammation.

## 1 INTRODUCTION

Asthma is the most prevalent chronic respiratory illness.^1^ Disease symptoms, such as wheezing and shortness of breath, are linked to airway hyperresponsiveness and variable obstruction, typically resulting from chronic airway inflammation.^2^, ^3^ Asthma is characterized by structural changes of the airways, including mucous metaplasia, smooth muscle hypertrophy, and increased extracellular matrix deposition, collectively known as airway remodeling.^4^, ^5^ These processes lead to increased stiffness and narrowing of asthmatic airways, resulting in obstructive ventilatory impairment.^6^ Studies in childhood asthma indicate that airway remodeling begins early and may persists into adulthood, suggesting it could be a primary pathogenic event not amenable to standard asthma care.^7^, ^8^, ^9^ On the other hand, advanced remodeling is also a common feature of severe asthma, with over half of the patients developing fixed airway obstruction (FAO).^10^, ^11^ In these cases, remodeling likely results from airway epithelial activation or injury due to environmental exposures and chronic inflammation, leading to aberrant repair and pro‐fibrotic signaling. These findings raise the question of to what extent remodeling occurs as a result of asthmatic inflammation, and whether it might proceed differently depending on the type of airway inflammation.

Over 2 decades ago, Wenzel et al.^12^ discovered inflammatory subtypes of severe asthma based on the presence of eosinophils in the bronchial mucosa. The subsequent description of the type‐2 (T2) endotype,^13^ characterized by overexpression of IL‐13‐stimulated genes and airway eosinophilia, has been crucial in understanding the diversity of asthma and paved the way for the development of new therapies. Though mechanisms of non‐T2 asthma are less understood, growing evidence suggests that some variants may be driven by type‐3 (T3) immune mechanisms associated with Th17 cells and IL‐17A signaling. Given that T2‐acting agents are currently a mainstay of treatment for severe uncontrolled asthma, understanding the mechanisms of remodeling in T2 and non‐T2 asthma is important as it may explain some disease‐modifying effects of these therapies.^14^ Whether certain inflammatory subtypes lead to more advanced airway remodeling is still debated.^15^ Some earlier studies suggested that signs of T2 immune activation in asthma correlated with greater airway obstruction,^16^, ^17^ but this was not replicated in other cohorts.^13^, ^18^ These inconsistencies may be partly related to various biomarkers of T2 asthma,^19^ including surrogate measures such as FeNO or blood eosinophils, or they may reflect the heterogeneity of remodeling itself.^20^ Additionally, previous research focused mostly on lung function decline using spirometry data without direct measurements of the airway wall.

Based on this background, we hypothesized that the progression of remodeling in severe asthma depends on the type of lower airway inflammation. Using mRNA signatures in the bronchial mucosa, we identified patients with different immunological endotypes in whom we assessed various parameters of airway remodeling and the magnitude of ventilatory impairment. In search for a mechanistic explanation, we also analyzed in vitro responses of bronchial epithelium to stimulation with key mediators of asthmatic inflammation.

## 2 RESULTS

### 2.1 Bronchial biopsy mRNA‐expression of inflammatory genes identifies T2‐high endotype of severe asthma

**FIGURE 1:**
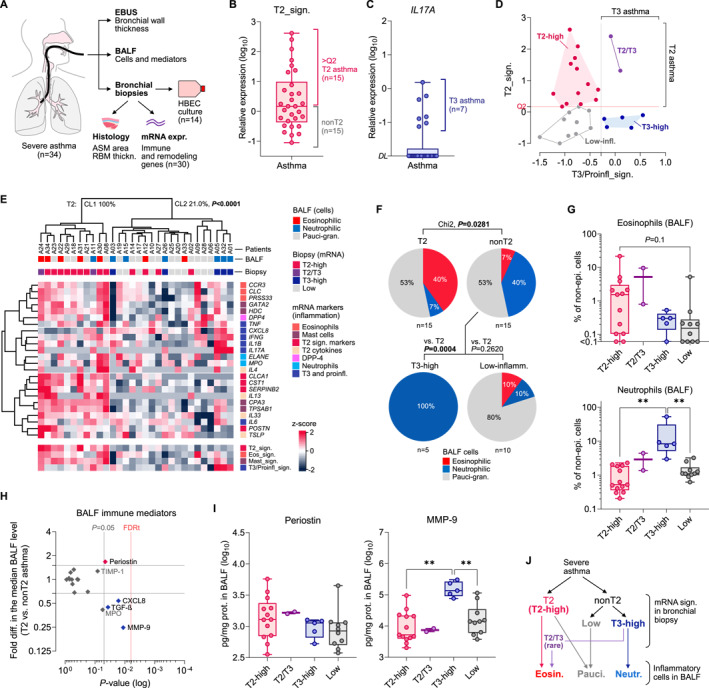
Bronchial biopsy mRNA signatures identify immune endotypes of severe asthma. (A) Study design. Endobronchial ultrasound (EBUS), bronchoalveolar lavage fluid (BALF), and bronchial biopsies were conducted in 34 severe asthmatics. mRNA expression was analyzed in 30 patients. ASM, airway smooth muscle; RBM, reticular basement membrane; HBEC, human bronchial epithelial cells. (B) Average expression of T2 signature genes and (C) *IL1*7A with cutoffs to identify patients with T2 and T3 asthma. DL, detection limit (D) Comparison of T2 and T3/proinfl. gene expression and distinguishing of immune endotypes. (E) Cluster classification (Euclidean dist.) and heat matrix of biopsy mRNA expression (immune genes, target‐centric) in individual asthma patients. Color legends indicate BALF inflammatory phenotypes, mRNA signatures, and key gene groups. The two patient clusters differed significantly with distribution of the T2 endotype (upper part). (F) Frequency of inflammatory phenotypes based on mRNA signature in bronchial biopsy. (G) Percentage of BALF eosinophils and neutrophils in patients with different immune endotypes. T2/T3 not analyzed due to low sample size. **p < 0.01 (Mann‐Whitney). (H) Fold difference in the BALF immune mediator levels in T2 asthma compared to nonT2. Vertical lines indicate significance thresholds (*p* = 0.05, Mann‐Whitney; FDRt, false discovery rate threshold at *q* = 0.1). Horizontal lines indicate 1.5‐fold difference. (I) BALF concentration of Periostin and MMP‐9 in the study subgroups. (J) Relationship between bronchial biopsy mRNA signatures and BALF inflammatory cells in severe asthma, as evidenced by this study.

We performed bronchoscopy in patients with severe asthma, directly measuring airway wall thickness with endobronchial ultrasound (EBUS) and sampling endobronchial biopsies for morphometry and gene expression analysis (Figure 1A). To characterize immune endotypes, we investigated the mRNA expression of immune genes, including canonical T2 markers (eg, *CST1*, *CLCA1*).^21^, ^22^, ^23^, ^24^ The expression of T2‐genes showed considerable variance across the samples, with a range exceeding 1000‐fold (Extended Data Figure S1a). Since healthy donors were not sampled, we were not able to provide normal ranges for T2 markers. Instead, we utilized the averaged expression score based on *CST1*, *CLCA1* and *SERPINB2*, referred to as the T2 signature (T2_sign, Extended Data Figure S1b–d) and next stratified patients as having T2 or nonT2 profiles based on the median T2_sign (Figure 1B). Seven patients showed detectable *IL1*7A transcripts and were classified as T3‐high (Figure 1C). Expression of *IL1*7A correlated with *IL6*, *IL1B* and *IFNG* (Extended Data Figure S2ab) confirming linkage with proinflammatory gene expression profile. To account for this relationship, in the analyses we also included the T3/Proinfl._sign., which was the average expression of *IL1*7A and top proinflammatory genes (Extended Data Figure S2c). When T2 and T3/Proinfl. signatures were contrasted (Figure 1D), we were able to distinguish pure T2‐high (*n* = 13), T3‐high (*n* = 5), mixed T2/T3 (*n* = 2), and low‐inflammatory subgroups (*n* = 10), with distribution similar to that observed in earlier studies on bronchial transcriptome in asthma.^18^, ^25^ Unsupervised analysis comprising all studied immune genes confirmed grouping of the majority of T2 samples (73.3%, Fisher *p* = 0.0025) as a separate cluster (Figure 1E).

Next, we compared clinical characteristics and bronchoalveolar lavage fluid (BALF) cells in patients with different endotypes. T2 and nonT2 patients showed similar clinical characteristics (Table 1), including disease duration (median 15 vs. 17 years, Mann‐Whitney *p* = 0.8779), atopy status (SPT+, 60 vs. 46%, Fisher *p* = 0.7152) and asthma treatment (eg, ICS, 1800 vs. 1920 μg/d, *p* = 0.6125). As expected, T2 patients were characterized by increased blood eosinophil count compared with nonT2 patients (383 vs. 112 cells/μL, *p* = 0.0032). Further subgroup analysis revealed increased blood neutrophil counts in T3‐high asthma (7.72 × 10^3^ vs. 4.65 × 10^3^ cells/μL in T2‐high, *p* = 0.0002), accompanied by increased serum CRP (12.5 vs. 1.3 mg/L in T2‐high, *p* = 0.0068) (Extended Data Table S3). However, clinical characteristics were comparable to those of T2‐high asthma (Extended Data Table S3).

**TABLE 1:** Clinical characteristics and BALF cell differential in asthma patients with T2 and nonT2 mRNA expression signatures in the bronchial biopsy.

	T2 (*n* = 15)	nonT2 (*n* = 15)	Median difference or odds ratio (95% CI)	*p*‐value
Age (years)	50 [42–54]	45 [40–57]	5 (−8, 8)	0.7982
Females (*n*, %)	8 (53.3%)	9 (60.0%)	0.76 (0.18, 2.95)	>0.9999
BMI (kg/m^2^)	25.7 [22.1–28.7]	24.5 [20.2–28.7]	1.2 (−3.0, 4.3)	0.5739
Age of onset (year)	32 [14–42]	26 [17–48]	6 [‐12, 15]	0.9267
Asthma duration (years)	15 [8–33]	17 [7–30]	−2 (−12, 8.5)	0.8779
Positive SPT (*n*, %)	9 (60.0%)	7 (46.1%)	1.71 (0.45, 7.28)	0.7152
ACT (score)	12 [9–14]	13 [11–18]	−1 (−6, 1)	0.3699
AQLQ (score)	3.31 [2.36–4.88]	4.16 [3.54–4.94]	−0.85 (−1.68, 0.47)	0.2470
Number of exacerbations (n/year)	4 [3–8]	5 [2–8]	−1 (−2, 3)	0.6435
ICS (μg/d)^1^	1800 [1000–2200]	1920 [1120–2400]	−120 (−800, 500)	0.6125
OCS (mg/d)^2^	4 [0–16]	8 [0–16]	−4 (−8, 4)	0.9380
OCS (*n*, %)	11 (73.3%)	8 (53.3%)	2.41 (0.57, 9.12)	0.4497
LABA (*n*, %)	15 (100%)	15 (100%)	n.a.	>0.9999
LAMA (*n*, %)	6 (40.0%)	6 (40.0%)	1 (0.24, 4.15)	>0.9999
SAMA (*n*, %)	5 (33.3%)	2 (13.3%)	3.25 (0.46, 18.3)	0.3898
LTRA (*n*, %)	4 (26.7%)	3 (20.0%)	1.46 (0.32, 6.73)	>0.9999
Blood eosinophils (cells/μL)	383 [152–482]	112 [30–173]	271 (54, 370)	**0.0032**
Blood basophils (cells/μL)	30 [18–69]	23 [17–37]	7 (−9, 38)	0.2854
Blood neutrophils (cells/μL)	4500 [3953–5609]	6830 [3064–7720]	−2329 (−2995, 842)	0.1736
Serum total IgE (IU/mL)	85.5 [39.9–174.5]	64.1 [17.8–508.9]	21.4 (−195, 80.7)	0.6507
Serum CRP (mg/L)	1.3 [1.1–2.1]	3.7 [1.8–10.7]	−2.4 (−4.0, 0)	**0.0486**
BALF analysis				
BALF cell count (10^3^ cells/mL)	116 [92–188]	160 [128–240]	−44 (−80, 12)	0.1130
Macrophages (% of non epi. cells)	92.5 [84.9–96.5]	90.8 [74.7–96.4]	1.7 (−4.0, 11.3)	0.7437
Neutrophils (% of non epi. cells)	0.63 [0.41–1.91]	1.40 [1.10–7.36]	−0.78 (−2.77, −0.26)	**0.0235**
Eosinophils (% of non epi. cells)	1.53 [0.10–3.86]	0.20 [<0.1–0.30]	1.33 (−2.0, 0)	**0.0328**
Lymphocytes (% of non epi. cells)	4.19 [1.13–6.99]	4.98 [2.01–15.0]	−0.79 (−9.81, 1.56)	0.2328
BALF eosinophils >2% (*n*, %)	6 (40%)	1 (6.7%)	9.33 (1.0, 113.4)	0.0801
BALF neutrophils >3% (*n*, %)	1 (6.7%)	6 (40%)	0.11 (0.01, 1.0)	0.0801
Airway inflammation, E/N/P (n)^3^	6/1/8	1/6/8	n.a.	**0.0281**

*Note*: References: 1, dose adjusted for fluticasone; 2, dose adjusted for methylprednisolone; 3, incidence of lower airway inflammatory phenotypes in asthma patients based of BALF cell analysis: E, eosinophilic (>2% eosinophils, ≤3% neutrophils); N, neutrophilic (≤2% eosinophils, >3% neutrophils); P, pauci‐granulocytic (≤2% eosinophils, ≤3% neutrophils). Statistics: Data are presented as medians [Q1‐Q3 quartiles]. Median differences with Mann‐Whitney statistics (for continuous data) or odds ratios with contingency table statistics (for categorical data; Fisher's exact test, Chi‐square for E/N/P distribution) are presented in separate columns. Significant differences (*p* < 0.05) are highlighted with a bold font.

Abbreviations: ACT, Asthma Control Test; AQLQ, Asthma Quality of Life Questionnaire; BALF, bronchoalveolar lavage fluid; BMI, body mass index; CRP, C‐reactive protein; ICS, inhaled corticosteroids; LABA, long‐acting β adrenoceptor agonists; LAMA, long‐acting muscarinic antagonists; LTRA, leukotriene receptor antagonists; n.a., non applicable; OCS, oral corticosteroids; SAMA, short‐acting muscarinic‐antagonists; SPT, skin prick tests.

BALF analysis confirmed increased eosinophilia in T2 asthma (1.53 vs. 0.2% in nonT2, *p* = 0.0328), with parallel decrease in neutrophils (Table 1), so that cell patterns differed significantly across identified endotypes (Figure 1F). We also documented an increase in the percentage of BALF neutrophils in T3‐high asthma compared with other endotypes (Figure 1G, Extended Data Table S3). BALF mediator profiles partly confirmed these results by showing elevated Periostin in T2 asthma, accompanied by lower levels of CXCL8 and MMP‐9. However, these changes did not reach significance after correction for multiple comparisons (Figure 1H), and Periostin appeared comparable across all endotypes (Figure 1I, left). T3‐high asthma remained strongly associated with renowned proinflammatory markers MMP‐9, CXCL8 and IL‐6 (Figure 1I, right; Extended Data Figure S3). In summary, the T2 gene expression profile in the endobronchial biopsy was associated with eosinophilic or pauci‐granulocytic inflammation, whereas the nonT2 profile included patients with T3‐high or low‐inflammatory signatures, who showed either neutrophilic or pauci‐granulocytic inflammation (Figure 1J).

### 2.2 More advanced airway structural changes in asthma patients with T2 gene expression signature in the bronchial biopsy

**FIGURE 2:**
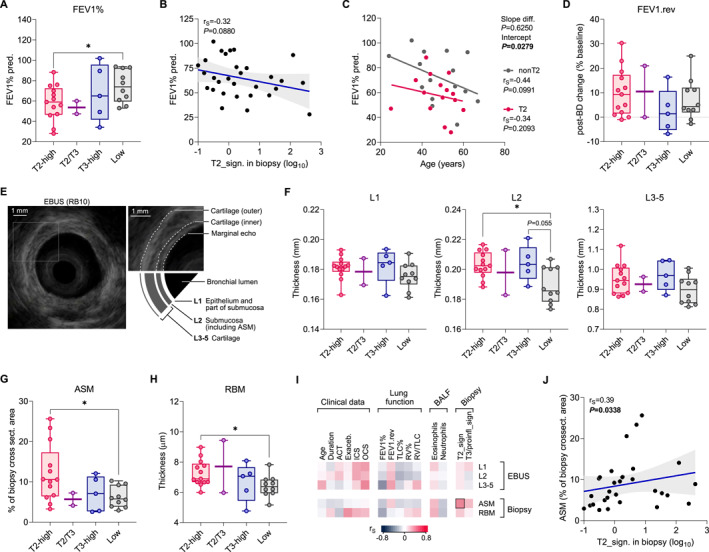
More advanced airway structural changes in asthma patients with increased expression of T2 genes in bronchial biopsy. (A) FEV1% predicted in asthma patients stratified by bronchial biopsy mRNA signature. (B) Correlation between T2 mRNA signature and FEV1% (*n* = 30; fitted regression line with 95%CI range). Spearman rank correlation coefficient (r_S_) and *p*‐value in upper left. (C) FEV1 decline with age in T2‐high versus T2‐low patients. Statistics show r_S_ and *p*‐values with slope (simple regression) and intercept difference. (D) No difference in post‐bronchodilator (BD) FEV1 change in asthma patients with different immune endotypes. (E) Representative endobronchial ultrasound image (EBUS) with 20 MHz radial probe located in bronchus RB10. Inset shows hyperechoic layer borders in the cartilaginous area. Schematic of bronchial wall layers (L1 to L5) identified by EBUS. (F) Average thickness of L1, L2 and combined L3‐5 bronchial wall layers. (G) Airway smooth muscle (ASM) mass expressed as the average cross‐sectional area in the bronchial biopsy histology. (H) Average reticular basement membrane (RBM) thickness in study groups. (I) Heat matrix summarizing correlation (r_S_, Spearman coefficient; all *p*‐values >0.01) of airway structural measures (EBUS and biopsy) with clinical variables, lung function, inflammatory cells in BALF, and mRNA signature in biopsy. ICS, inhaled corticosteroids; OCS, oral corticosteroids; TLC, total lung capacity; RV, residual volume. (J) Weak positive correlation between bronchial biopsy ASM area and T2 gene expression. Statistics in (a), (d), (f‐h): Data shown as medians and quartiles. Mann‐Whitney: *p < 0.05. T2/T3 patient data are shown but not included in analysis due to low sample size.

Subsequently, we investigated whether bronchial mRNA expression profiles affect lung function and airway structural measures. Patients with T2 profile showed comparable static lung volumes, FEV1/FVC ratio, and similar prevalence of FAO compared to nonT2 (Table 2). Interestingly, they demonstrated decreased FEV1 compared to non‐T2 (median 59.0 v 73.9% predicted, *p* = 0.0419), indicating more pronounced airway obstruction. Subgroup analysis revealed that this difference held significance when comparing T2‐high and low‐inflammatory asthma (Figure 2A, Extended Data Table S4). The expression of T2‐associated genes in bronchial biopsy weakly negatively correlated with FEV1% (Figure 2B), although it did not reach statistical significance (Spearman r_S_ = −0.32, *p* = 0.088). The rate of FEV1 decline with age was not pronounced in T2 asthma, despite a significant difference in *Y*‐intercept when compared with nonT2 asthma (Figure 2C). The reversibility of FEV1 after inhalation of bronchodilator was comparable in T2 and nonT2 asthma (Table 2), and did not show a significant decline in the T3 variant either (Figure 2D).

**TABLE 2:** Lung function and airway structural measures in asthma patients with T2 and nonT2 immune signatures in the bronchial biopsy.

	T2 (*n* = 15)	nonT2 (*n* = 15)	Median difference or odds ratio (95% CI)	*p*‐value
Lung function				
FVC (% predicted)	77.0 [67.0–89.0]	86.5 [76.0–97.0]	−9.5 (−21.1, 3.0)	0.1370
FEV1 (% predicted)	59.0 [47.1–70.0]	70.0 [54.8–92.5]	−11.0 (−30.5, −0.8)	**0.0419**
FEV1%FVC before BD	59.0 [50.3–71.9]	69.1 [61.3–74.3]	−10.1 (−16.2, 2.32)	0.1607
FEV1%FVC after BD	64.6 [54.0–77.6]	71.2 [62.7–73.9]	−6.6 (−11.9, 7.43)	0.8702
FAO (*n*, %)	8 (53.3%)	6 (40.0%)	1.71 (0.45, 7.28)	0.7152
FEV1.rev (% change after BD)	9.3 [1.0–18.6]	3.7 [‐1.0‐11.2]	5.6 (−2.9, 11.8)	0.2623
TLC (% predicted)	100.0 [89.9–117.9]	110.9 [102.2–124.0]	−10.9 (−22.0, 5.4)	0.1873
RV (% predicted)	121.0 [102.0–2.2.0]	135.3 [124.5–177.0]	−14.3 (−47.0, 33.1)	0.2806
RV/TLC (ratio)	0.44 [0.37–0.47]	0.43 [0.38–0.55]	0.01 (−0.11, 0.06)	0.6529
EBUS measurements				
L1 (mm)	0.182 [0.177–0.187]	0.177 [0.172–0.185]	0.005 (−0.003, 0.010)	0.5125
L2 (mm)	0.203 [0.196–0.213]	0.199 [0.184–0.203]	0.004 (−0.001, 0.017)	0.1160
L3‐5 (mm)	0.945 [0.882–0.999]	0.930 [0.840–0.989]	0.014 (−0.044, 0.083)	0.3892
Bronchial biopsy histology				
ASM (% of biopsy cross‐section area)	10.08 [6.16–14.09]	5.97 [3.67–9.56]	4.11 (0.22, 7.67)	**0.0295**
RBM thickness (μm)	6.89 [6.66–8.02]	6.55 [5.95–7.06]	0.34 (0.01, 1.57)	**0.0453**

*Note*: Statistics: Data are presented as medians [Q1‐Q3 quartiles]. Median differences with Mann‐Whitney statistics (for continuous data) or odds ratio with contingency table statistics (only for FAO; Fisher's exact test) are presented in separate columns. Significant differences (*p* < 0.05) are highlighted with a bold font.

Abbreviations: ASM, airway smooth muscle; BD, bronchodilator (short acting β2‐agonist); FAO, fixed airway obstruction (defined as FEV1%FVC post BD <70%); FEV1, forced expiratory volume in 1 s; FEV1.rev, reversibility of FEV1; FVC, forced vital capacity; RBM, reticular basement membrane; RV, residual volume; TLC, total lung capacity.

To investigate whether inflammatory endotypes are associated with airway structural changes, we measured bronchial wall thickness with EBUS, and assessed bronchial biopsy morphometry. The radial EBUS provides a circular view of the multi‐layered bronchial wall (Figure 2E), including the important L2 layer representing the smooth muscle of the submucosa.^26^, ^27^ Initial comparison T2 or nonT2 asthma did not reveal significant differences (Table 2), and no correlation with immune or cellular mRNA signatures (eg, for L2 and T2 signature: *r* = 0.07, *p* = 0.71). Further analysis revealed heterogeneity within the nonT2 group (Figure 2F), as patients with low‐inflammatory endotype exhibited a significant decrease in L2 thickness compared to T2‐high patients (Mann‐Whitney *p* = 0.0178) and a trend when compared to T3‐high patients (*p* = 0.0553). Although T2‐high and T3‐high patients showed similar L2 thicknesses, the results were only ∼10% higher than those of low‐inflammatory asthma (Extended Data Table S4).

The airway smooth muscle (ASM) mass was increased (median 1.7‐fold) in T2 asthma compared with nonT2 asthma (Table 2; 6.0 vs. 10.1%, *p* = 0.0295). Interestingly, ASM content was notably lower in patients with low‐inflammatory profile compared with T2‐high asthma (5.8 vs. 10.6%, *p* = 0.0178), although it did not differ from T3‐high asthma (Figure 2G; Extended Data Table S4). The T2‐high group showed marked diversity in ASM content (Figure 2G), with only five patients having values exceeding 90‐percentile of the nonT2 group (i.e., ASM area >11%). However, these patients did not exhibit significant clinical differences (not shown). We also recorded small, but significant increase in reticular basement (RBM) thickness in T2‐high patients compared to low‐inflammatory asthma (by 8%, 6.89 vs. 6.38 μm, *p* = 0.0453) but not when compared to T3‐high asthma (Figure 2H). Finally, correlation of clinical and immunological data with structural measures revealed mostly weak associations (Figure 2I; all coefficients <0.4, *p* > 0.01). Of these, the T2 signature in the bronchial biopsy weakly positively correlated with ASM mass (Figure 2J; *r*_S_ = 0.39, *p* = 0.0338) but not with RBM thickness (*r*_S_ = 0.17), or L2 (*r*_S_ = 0.07). Clinical parameters did not correlate with biopsy morphometry, except for a weak association between RBM thickness and number of asthma exacerbations (*r*_S_ = 0.36, *p* = 0.0375). Altogether, these data suggest that severe asthma patients showing increased expression of T2 genes in bronchial mucosa are characterized by more advanced structural changes in the airways.

### 2.3 Eosinophil markers in bronchial biopsy correlate with expression of remodeling genes

**FIGURE 3:**
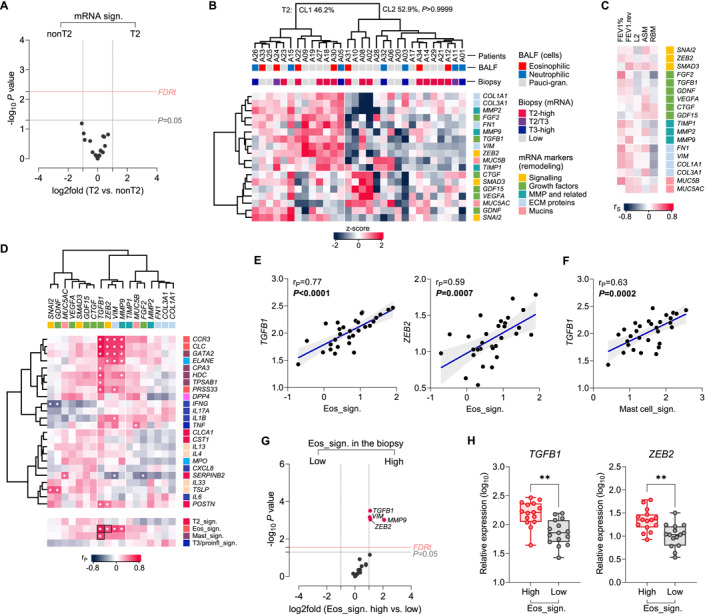
Eosinophil markers in the bronchial biopsy are associated with increased expression of remodeling genes. (A) No difference in relative mRNA expression of remodeling genes comparing asthma patients with high or low expression of T2 genes. 2‐sided *t*‐test; FDRt, false discovery rate threshold at *q* = 0.1. (B) Cluster classification (Euclidean dist.) and matrix of relative mRNA expression of remodeling genes (target‐centric) in individual asthma patients. BALF inflammatory phenotypes, T2 and T3 signatures, and key gene groups are highlighted with colors. No difference in the frequency of T2 endotype in the two patient clusters (upper part, Fisher test). (C) Heat map showing weak correlation (r_S_, Spearman coefficient, all *p*‐values >0.01) of important functional and structural measures of remodeling with mRNA expression of remodeling genes in bronchial biopsy. FEV1, forced expiratory volume in 1 s; ASM, airway smooth muscle; RBM, reticular basement membrane. (D) Heat matrix of correlation (r_P_, Pearson coefficient, *p < 0.01) between expression of remodeling (top) and immune genes (side). (E) Scatter plots showing marked positive correlation between eosinophil mRNA signature and expression of *TGFB1* and *ZEB2*, and (F) between mast cell mRNA signature and *TGFB1*. Pearson coefficients and *p*‐values in upper left. (G) Only four remodeling genes (labeled) were significantly increased in patients with high expression of eosinophil marker genes in bronchial biopsy (stratified by median). 2‐sided *t*‐test; FDRt, *q* = 0.1. (H) Expression of *TGFB1* and *ZEB2* in Eos_sign. high and low subgroups. Mann‐Whitney: **p < 0.01.

Next, we searched for a potential mechanism linking T2 asthma and airway remodeling. To this end, we initially compared the expression of remodeling genes in bronchial biopsies, including important growth factors (eg, *TGFB1*, *FGF2*, and *CTGF*), transcription factors (eg, *SNAI2* and *ZEB2*), and extracellular matrix components (eg, *COL1A1* and *VIM*). Surprisingly, the initial comparison of T2 and nonT2 patients did not show any difference in mRNA expression of remodeling genes (Figure 3A). We observed marked heterogeneity in remodeling gene expression profile across the studied samples (Figure 3B), but the distribution of T2 samples was similar in the identified patient clusters. T3‐high patients also did not show any specific pattern (not shown). Consistent with these findings, there was no clear association between expression of remodeling genes in bronchial biopsies and lung function or structural measures of remodeling (Figure 3C).

Interestingly, cross‐correlation of remodeling and inflammatory gene expression data revealed a subset of genes strongly associated with eosinophil markers (Figure 3D). In particular, the eosinophil mRNA signature clearly positively correlated with the expression of *TGFB1* encoding TGF‐β1 (Figure 3E; Pearsons *r* = 0.77, *p* < 0.0001). TGF‐β1 is a potent inducer of various remodeling genes in bronchial epithelium, involved in processes of epithelial‐mesenchymal transition (EMT) and fibrosis. Another gene linked with eosinophil signature was *ZEB2* (*r* = 0.59, *p* = 0.0007), encoding a key transcription factor regulating the EMT pathway. We also showed a linkage with the expression of *VIM* (*r* = 0.64, *p* = 0.0001) encoding vimentin, and *MMP9* (*r* = 0.57, *p* = 0.0011) encoding matrix metalloproteinase‐9. A similar pattern was observed for mast cell mRNA signature, which also showed positive correlation with *TGFB1* (Figure 3F; Pearsons *r* = 0.63, *p* = 0.0002), *ZEB2*, *VIM* and *MMP9* (*r* = 0.4 for all). Expression of these four genes was also significantly increased (∼2 to 4‐fold) in asthma patients characterized by high expression of eosinophil mRNA markers in bronchial biopsy (Figure 3G,H). These data suggest that bronchial expression of key remodeling factors in severe asthma is associated with eosinophilic infiltration in the bronchial mucosa.

### 2.4 Similar expression of remodeling genes and response to TGF‐β1 in bronchial epithelial cells derived from patients with T2 and nonT2 asthma

**FIGURE 4:**
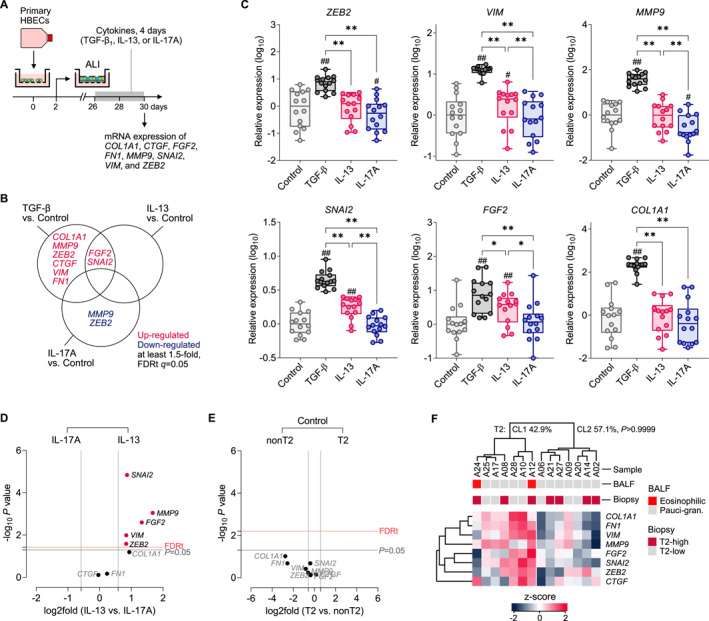
Similar expression of remodeling genes and TGF‐β_1_ response in bronchial epithelial cells from T2 and nonT2 patients. (A) Expression of key remodeling genes was assessed by qPCR in air‐liquid interface (ALI)‐differentiated HBECs exposed to TGF‐β_1_, IL‐13, or IL‐17A (*n* = 14). (B) Differentially expressed genes in various cytokine conditions (10 ng/mL each) compared to control. FDRt, false discovery rate threshold. (C) mRNA expression (relative to control) of remodeling genes in HBECs incubated with cytokines. Data shown as medians with quartiles. RM‐ANOVA (Tukey): #*p* < 0.05, ##*p* < 0.01 compared to control. *p < 0.05, **p < 0.01 as assigned. (D) Relative expression difference in cells treated with IL‐13 compared to IL‐17A (*n* = 14). Genes significantly upregulated under IL‐13 conditions are marked *red*. 2‐sided paired *t*‐test, FDRt *q* = 0.05. Vertical lines indicate 1.5‐fold difference (E) Similar expression of remodeling genes in ALI‐grown HBECs from T2 and nonT2 patients (control conditions). 2‐sided *t*‐test. (F) Cluster classification (Euclidean distance) and heat matrix of expression of remodeling genes (target centric) in ALI‐grown HBECs (control conditions). No association with T2 airway inflammation in the donor's airways (upper part, Fisher test).

Next, we investigated whether the bronchial epithelial response to TGF‐β1 is more pronounced in cells isolated from patients with T2 asthma. We also stimulated cells with key effector cytokines of T2 (IL‐13) and nonT2 (IL‐17A) asthma. Human bronchial epithelial cells (HBECs) derived from T2 (*n* = 7) or nonT2 (*n* = 7) patients were mucociliary differentiated in vitro, next exposed to TGF‐β1, IL‐13, or IL‐17A (4 days, 10 ng/mL each), and harvested to determine mRNA expression of remodeling genes (Figure 4A). Exposure to TGF‐β1 resulted in a dose‐dependent upregulation of all the genes included in the panel (Extended data Figure S4a,b). We also noticed a significant increase in the expression of *FGF2* and *SNAI2* in cells incubated with IL‐13 (Figure 4B,C, Extended Data Figure S4c). In contrast, exposure to IL‐17A resulted in decreased epithelial expression of *ZEB2* and *MMP9*, and a trend toward decrease in *COL1A1* (Extended Data Figure S4d). Interestingly, most remodeling markers were significantly increased in IL‐13 conditions (top *SNAI2*, *MMP9*, and *FGF2*) if directly compared to IL‐17A, indicating a contrasting response depending on the prevailing immune profile (Figure 4D).

We observed considerable heterogeneity in the baseline expression of remodeling genes; however, HBECs derived from patients with T2 asthma did not differ compared with nonT2 (Figure 4E). The expression of genes in response to TGF‐β1 was also comparable between the two endotypes (Extended Data Figure S5). Cluster classification revealed a subgroup of patient samples showing an increased remodeling mRNA signature in cultured HBECs (CL1 in Figure 4F), but these were derived equally from both T2 and nonT2 patients (Fisher test, *p* > 0.9999). Moreover, increased expression of remodeling genes in cultured HBECs was not associated with clinical characteristic of the donor patients nor with more advanced structural remodeling (Extended Data Table S5). Altogether, these data indicate that bronchial epithelial cells collected from asthma patients with T2 airway signature do not differ intrinsically with remodeling gene expression and response to TGF‐β1 compared to nonT2 patients. However, stimulation with T2 cytokines may result in pro‐fibrotic state in the bronchial epithelium.

### 2.5 Blood eosinophilia is a good predictor of bronchial T2 immune signature in severe asthma

**FIGURE 5:**
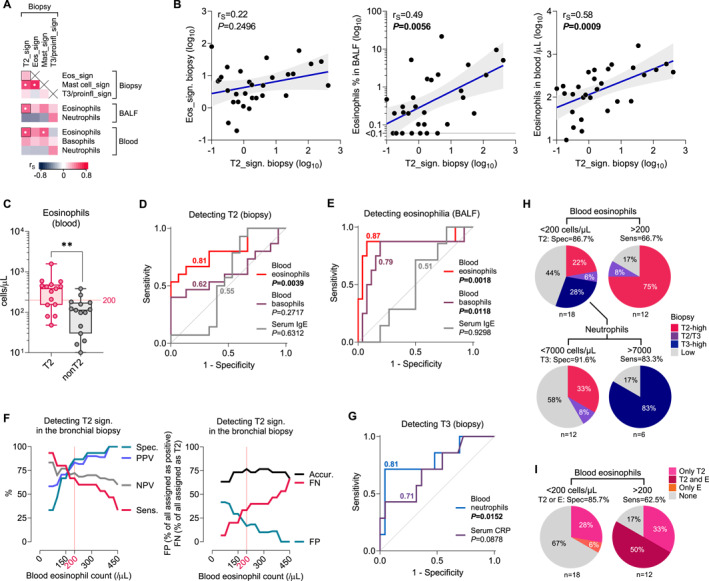
Blood eosinophil count is a good predictor of bronchial biopsy T2‐signature in severe asthma. (A) Correlation (r_S_, Spearman; *p < 0.01) of bronchial biopsy mRNA signatures with BALF and blood cells. (B) Scatter plots showing correlation between T2 mRNA signature in the bronchial biopsy and various measures of eosinophilia in the airway (i.e., eosinophil signature in the biopsy [left], percentage of BALF eosinophils [middle]) and blood [right]). Spearman coefficients and *p*‐values in upper left. (C) Blood eosinophil counts in patients with T2 and nonT2 signature in bronchial biopsy. Mann‐Whitney: **p < 0.01. (D) Receiver operating characteristic (ROC) curves of blood eosinophil and basophil counts, and serum IgE in detection of T2 signature in bronchial biopsy. Numeric values represent areas under the ROC curve. (E) ROC performance in predicting BALF eosinophilia >2%. (F) Diagnostic test performance of blood eosinophil counts in the detection of T2 signature in bronchi. Sens., sensitivity; Spec., specificity; PPV, positive predictive value; NPV, negative predictive value; Accur., accuracy; FN, false negative; FP, false positive. (G) ROC performance of blood neutrophil count and serum C‐reactive protein (CRP) in predicting T3 signature in bronchial biopsy. (H) Stratification of patients based on blood eosinophil count cutoff 200 cells/μL (upper part; *n* = 30) and neutrophil count cutoff 7000 cells/μL (lower part, *n* = 18, only patients with blood eosinophilia <200 cells/μL included). (I) Prevalence of T2 signature in the biopsy and/or eosinophilia in BALF (labelled E) in patients stratified based on blood eosinophil count.

In the final part of the study, we attempted to determine if blood eosinophil count, a primary surrogate marker of eosinophilic inflammation in asthma, would be sensitive enough to detect T2 immune signature in the bronchi. First, we analyzed the correlation of bronchial biopsy immune signatures with airway and systemic eosinophilia (Figure 5A). Surprisingly, expression of T2 genes was poorly correlated with eosinophil signature (Figure 5B; Spearman *r* = 0.22, *p* = 0.2496), suggesting that mucosal eosinophils are not always detected in patients with T2 signature. Interestingly, there was a significant positive correlation of T2 signature with BALF (*r* = 0.49, *p* = 0.0056) and particularly blood eosinophils (Figure 5B, right; *r* = 0.58, *p* = 0.0009), implying that blood eosinophil count may be a good predictor of airway immune signature. Indeed, the median number of blood eosinophils was 3.4‐fold higher in T2 asthma compared with nonT2 asthma (Figure 5C; 383 vs. 112 cells/μL, Mann‐Whitney *p* = 0.0032).

We next assessed the diagnostic utility of blood surrogate markers in detecting different types of airway inflammation. Blood eosinophil count showed excellent diagnostic performance in detecting patients with T2 signature (AUC = 0.81, *p* = 0.0039) compared with blood basophils and serum IgE (Figure 5D). Blood eosinophil count also performed well in the detection of patients with BALF eosinophilia (Figure 5E; AUC = 0.87, *p* = 0.0018). In search for blood eosinophil count values useful to distinguish T2 asthma, we determined diagnostic test utility measures at different cutoffs (Figure 5F, left). It turned out that eosinophilia 200 cells/μL showed optimal performance (sensitivity, 66.6%; specificity, 86.7%; PPV, 83.3%; NPV, 72.2%), and was able to detect patients with T2 asthma with 76.7% accuracy. For value 150 cells/μL we showed increase in sensitivity (sensitivity, 80%; specificity, 60%; PPV, 66.7%; NPV, 75%) at the cost of decreased accuracy (70%). Using 200 cells/μL cutoff, 33.3% of patients with T2 signature were falsely assigned as nonT2, with only 16.7% of false positive results (Figure 5F, right). Decreasing cutoff to 150 cells/μL results in 20% false negative and 33.3% false positive results. Interestingly, blood neutrophil count showed excellent performance (AUC = 0.81, *p* = 0.0152) and serum CRP showed moderate performance (AUC = 0.71, *p* = 0.0878) in detecting T3 signature in the bronchial biopsy (Figure 5G); however, these data are less credible due to low sample size. To summarize, with eosinophil 200 cells/μL and neutrophil 7000 cells/μL cutoffs, we were able to correctly assign 76.7% of patients as T2 or nonT2, and 83.3% patients as T3 or nonT3 (Figure 5H), with an overall 75% correct assignment as T2‐high, T3‐high, or low‐inflammatory. Blood eosinophil count was also acceptable (AUC = 0.76, *p* = 0.0159) in the detection of patients who had either a T2 immune signature or increased airway eosinophilia (Extended Data Figure S6), so that 200 cells/μL cutoff showed 62.5% sensitivity and 85.7% specificity, with a correct assignment of 73.3% patients (Figure 5I).

## 3 DISCUSSION

The discovery of the immunological heterogeneity of asthma has spurred numerous studies aimed at understanding how the immune mechanisms of the disease translate into clinical phenotypes. Still, a significant research challenge remains in explaining the contribution of airway inflammation to the progression of remodeling in severe disease. To address this, we conducted an observational study examining the bronchial expression of remodeling‐related genes in patients with severe asthma stratified according to their bronchial inflammatory signatures. We also had the unique opportunity to simultaneously measure the thickness of the airway walls and directly assess key morphometric parameters in the bronchi of asthmatic patients. Our data suggest that T2 inflammation is associated with more advanced airway remodeling and greater bronchial obstruction. The proposed mechanisms for these changes involve increased expression of pro‐remodeling genes associated with uncontrolled eosinophilic inflammation and exposure to T2‐related cytokines.

### 3.1 More advanced airway remodeling in T2‐high endotype of severe asthma

The initial comparison revealed comparable clinical characteristics of T2 and nonT2 asthma, including similar duration of the disease, symptom burden, and steroid dose. Both groups showed airway obstruction with only partial reversibility to the bronchodilator. However, the predicted FEV1 values were significantly lower in patients with increased expression of T2 genes. Our results are in line with earlier studies describing a greater decline in lung function in T2 asthma, in particular with the multi‐omics analysis of the U‐BIOPRED cohort. For example, cluster analysis of bronchial mucosa and sputum transcriptome data revealed a subgroup of patients characterized by a marked T2 signature, who demonstrated decreased FEV1.^16^, ^28^ Similarly, persistent airway limitation in severe asthma patients included in the U‐BIOPRED cohort was associated with distinct immune pathways, including IL‐13 signaling related to T2 immunity.^29^ Recently published large population study confirmed that the presence of T2 inflammation, reflected by elevated blood eosinophil count or FeNO, was associated with an accelerated decline of FEV1 with age, particularly in more severe asthma patients who already developed persistent airflow limitation.^17^ However, some studies that identified asthma endotypes by analyzing the bronchial brush transcriptome did not show decreased FEV1 in T2 asthma. For example, Woodruff et al.^13^ described comparable disease severity, lung function, and bronchodilator response in Th2‐high and Th2‐low asthma. Similarly, Choy et al.^18^ observed nearly identical FEV1% predicted in asthma endotypes identified by expression of either Th2‐ or Th17‐related genes. One reason for inconsistent results could be the differences in asthma severity and treatment among enrolled patients, with some studies focusing solely on ICS‐free mild asthma^13^ or including a wide range of mild to severe asthmatics.^18^ Furthermore, some of the discrepancies can be attributed to the generally low number of participants in studies involving bronchial sampling, which is also a limitation of the current project.

The novel aspect of our study is demonstrating concordant changes related to more advanced remodeling in T2 asthma. These changes were evident in the mucosa histology, the structure of the bronchial wall layers, and lung function tests. Specifically, patients with T2 asthma exhibited a nearly two‐fold increase in ASM area in bronchial biopsy specimens compared with the low‐inflammatory endotype, suggesting ASM hypertrophy. Additionally, these patients showed an increase in RBM thickness, though relative differences were much smaller compared to changes in ASM mass. Particularly interesting were EBUS data showing a significant increase in the thickness of the L2 layer, representing submucosa with ASM,^26^, ^27^, ^30^ yet only in comparison of T2‐high and low‐inflammatory asthma. The differences in L2 thickness were, however, quite small compared to ASM histology data, and there was no significant correlation between EBUS data and immune signatures. Our data are consistent with basic research confirming that T2 cytokines induce phenotype changes of ASM cells resulting in enhanced migration and contractility.^31^, ^32^, ^33^ Nevertheless, clinical data comparing direct measurements of ASM mass with transcriptomic signatures of severe asthma are scarce, and we are not aware of any EBUS study on this subject. The most related study by Kuo et al.^34^ showed no difference in ASM biopsy area in severe asthma patients with high or low eosinophil signatures in the bronchial mucosa.

Intriguingly, we observed a trend toward greater L2 thickness also in patients with T3‐high endotype, suggesting that L2 is generally more pronounced in patients with overt airway inflammation. T3‐high asthma is associated with neutrophilic airway inflammation, poor response to steroids, and marked pro‐inflammatory signature in the bronchi.^18^, ^25^ Notably, this rare asthma variant has been linked with more severe disease and airway obstruction,^35^, ^36^ also suggesting the contribution of IL‐17A‐axis cytokines to airway remodeling.^37^ Unfortunately, due to the low sample size, we were not able to credibly investigate the association between T3‐high variant and lung function or airway structural measures. However, some of our data suggest more advanced changes in this variant. For example, the T3‐high group showed poor reversibility of FEV1 (median 1.4% vs. 9.3% in T2‐high) and the highest prevalence of FAO (60% vs. 46.2% in T2‐high, and 30% in low‐inflammatory endotype), indicating that T2 and nonT2 mechanisms may contribute independently to remodeling and FEV1 decline, as previously demonstrated.^38^ These data collectively suggest that increased ASM layer, likely related to both ASM hypertrophy and increased propensity to constriction,^20^ is likely the factor more specific to T2 signature, while other changes such as bronchial wall thickness or lung function decline are more related to uncontrolled inflammation, regardless of whether it is driven by T2 or nonT2 mechanisms.

### 3.2 Airway eosinophilia associated with increased expression of TGF‐β regulated genes

In the next part of the study, we attempted to explain the mechanism by which T2 inflammation could promote structural remodeling of the airways. To this end, we assessed bronchial expression of an array of remodeling genes, including important growth factors, matrix components, and transcription factors. Unexpectedly, patients with T2 asthma did not show pro‐fibrotic gene expression profile, and there was also no association with certain clinical patterns. The only finding that caught our attention was a strong positive correlation between eosinophilic markers in the biopsy and expression of a subset of remodeling genes, which included *TGFB1*, encoding growth factor TGF‐β1, and *ZEB2*, encoding transcription factor crucial for processes of EMT. This result implicated that the presence of eosinophils in the bronchial mucosa could initiate a gene expression profile that promotes airway remodeling. Our results are consistent with previous research showing that eosinophils were able to induce production of TGF‐β1, thereby triggering processes of mesenchymal transition in bronchial epithelium,^39^ as well as upregulation of remodeling genes in smooth muscle cells and lung fibroblasts.^40^ Moreover, histological studies in asthma revealed that activated eosinophils in the submucosa themselves constitute a significant source of TGF‐β.^41^, ^42^, ^43^ Because TGF‐β1 is widely recognized as a master regulator of fibrosis and EMT in asthma,^44^ these results suggest altogether that eosinophils infiltrating the bronchial mucosa contribute to local production of TGF‐β1 and may induce pro‐fibrotic phenotype of the airway epithelium. But is it the intrinsic feature of airway epithelium that could respond differentially to pro‐fibrotic stimulation depending on asthma endotype? To verify this hypothesis, we performed an in vitro experiment using cultures of HBECs from T2 and nonT2 asthma patients. As expected, TGF‐β1 stimulation led to a significant increase in the expression of remodeling genes, but there was no difference related to asthma endotype. Of note, we observed contrasting effects of inflammatory cytokines, as stimulation with IL‐13, a canonical T2 cytokine, resulted in increased expression of key remodeling genes, whereas IL‐17A treatment showed mostly opposite effects. This result is in line with an earlier transcriptomic study showing a positive correlation between expression of TGF‐β1 and other members remodeling pathway with the Th2 signature in bronchial mucosa,^22^ thus explaining reports on increased bronchial fibrosis in T2 asthma.^13^

**FIGURE 6:**
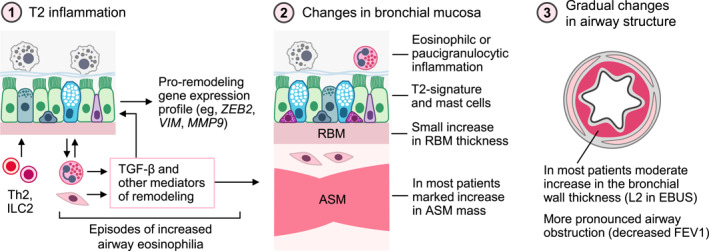
Summary of the key results of the study. (1) T2‐mediated inflammation is associated with episodes of airway eosinophilia, increased expression of TGF‐β, and other pro‐remodeling genes. (2) Uncontrolled airway inflammation may lead to local changes in bronchial mucosa, such as increased airway smooth muscle (ASM) mass and thickening of the reticular basement membrane (RBM). (3) Gradual progression of remodeling leads to permanent changes in the bronchial wall and greater obstructive ventilatory impairment.

Contribution of eosinophilic inflammation to airway remodeling would be a neat explanation for more extensive structural changes in T2‐high asthma, if it weren't for the fact that the T2 endotype does not always present with airway eosinophilia. Actually, in our study, more than half of the T2 patients had pauci‐granulocytic asthma. This suggests that the bronchial T2 signature in severe asthma could be more stable compared with airway eosinophilia. Similar fluctuations of inflammatory markers over time have already been reported in asthma.^19^, ^45^ Therefore, increased TGF‐β1 stimulation with a cascade of pro‐remodeling responses should also occur temporarily only in the presence of uncontrolled inflammation. Consequently, airway structural changes may accumulate gradually in response to these periods of greater immune activation, which in turn results in progression of airway obstruction (as summarized in Figure 6). Interestingly, clinical data showed that frequent asthma exacerbations, related by definition to periods of increased airway inflammation, were also associated with faster lung function decline particularly in severe eosinophilic asthma.^46^, ^47^, ^48^, ^49^ Unfortunately, the main limitation of our study is examining airway inflammation and structure at a single time‐point, representing only a snapshot over the variable course of the disease. However, repeating bronchoscopy with bronchial sampling just to explain whether the pro‐remodeling signature fluctuates alongside eosinophilia is not feasible due to ethical constraints.

### 3.3 Blood eosinophilia as a good surrogate marker of T2 asthma

As already pointed out, we observed a discrepancy between the expression of T2 genes in bronchial biopsies and eosinophilic inflammation. Earlier studies in severe asthma also reported only a weak association between T2 signature in bronchial mucosa and mucosal eosinophils.^18^, ^50^ Only a few reports compared the numbers of eosinophils in biopsies and BALF, also showing a rather weak correlation.^51^, ^52^ These differences are likely related to sampling of different airway compartments,^53^ shifts related to treatment modifications,^21^ or just the intrinsic variability, as evidenced by sputum transcriptomics data in the U‐BIOPRED cohort.^54^ These data prompted us to investigate whether blood eosinophil count, an established surrogate marker of eosinophilic inflammation, would reliably detect asthma patients with the T2 endotype. As it turned out, blood eosinophilia performed very well as a marker of T2 immune signature in bronchial mucosa (AUC = 0.81); however, it was less sensitive (AUC = 0.76) for detecting patients with either a T2 profile or airway eosinophilia. Our results also confirm the usefulness of lower cutoffs of blood eosinophil counts if they were used as inclusion criteria for T2‐targeted therapies.

### 3.4 Conclusions

Our data indicate that bronchial structural changes were more advanced in patients with severe asthma, showing a T2 gene signature in the bronchial mucosa. Further analysis revealed that expression of key remodeling genes was strongly associated with signs of tissue eosinophilia, suggesting that remodeling pathways in T2‐asthma could be driven by uncontrolled eosinophilic inflammation. Moreover, the lack of a clear linkage between Th17‐related markers and structural changes, along with the somewhat opposite effects of IL‐13 and IL‐17A in cultured bronchial epithelial cells, suggests that processes of airway remodeling induced by chronic inflammation are differentially regulated depending on the prevailing asthma endotype. Considerable inflammatory heterogeneity of severe asthma requires further studies to understand the distinctive mechanism of lung function decline, especially in T2‐low disease variants.

## 4 METHODS

For this observational, cross‐sectional study, we enrolled 34 patients with confirmed diagnosis of severe asthma based on Global Initiative for Asthma guidelines.^2^ In all subjects, we performed bronchoscopy with EBUS of the right lower lobe segmental bronchi, bronchoalveolar lavage (to quantify inflammatory cells), and bronchial biopsy sampling (Figure 1A). Bronchial biopsy specimens were used in histological studies and RNA isolation (*n* = 30). Additionally, we isolated and cryopreserved HBECs for in vitro experiments (*n* = 14). The study was conducted in accordance with the Declaration of Helsinki and its revisions. The study protocol was approved by the Jagiellonian University Bioethics Committee (KBET 122.6120.167.2015), and written informed consent was obtained from all participants.

Detailed methods are presented in the Online Repository. In brief, mRNA expression of inflammatory and remodeling genes in endobronchial biopsies was analyzed by TaqMan real‐time PCR (assays listed in Extended Data Table S1) using QuantStudio 12K Flex System (Applied Biosystems, Foster City, CA). BALF mediators were evaluated by ELISA or Luminex (listed in Extended Data Table S2) using Magpix equipment (Luminex Corp., Austin, TX). Bronchial biopsy morphometry included ASM content using α‐smooth muscle actin immunostaining (Dako Denmark A/S, Glostrup, Denmark), and measurements of RBM thickness. HBEC cultures were used to analyze cytokine responses. Mucociliary differentiated epithelia were exposed chronically (4 days) to TGF‐β1, IL‐13 or IL‐17A and then harvested for mRNA expression analysis.

## AUTHOR CONTRIBUTIONS

**Bogdan Jakiela**: Conceptualization; investigation; writing—original draft; data curation; formal analysis; visualization; methodology; supervision. **Karolina Gorka**: Writing—review and editing; resources. **Iwona Gross‐Sondej**: Writing—review and editing; resources. **Slawomir Mikrut**: Methodology; investigation; software; writing—review and editing. **Krzysztof Okon**: Investigation; writing—review and editing; formal analysis. **Piotr Sadowski**: Investigation; writing—review and editing; formal analysis. **Anna Andrychiewicz**: Writing—review and editing; methodology. **Hanna Plutecka**: Writing—review and editing; methodology. **Tomasz Stachura**: Resources; writing—review and editing. **Grazyna Bochenek**: Resources; writing—review and editing; investigation. **Stanislawa Bazan‐Socha**: Resources; writing—review and editing. **Krzysztof Sladek**: Resources; investigation; writing—review and editing. **Jerzy Soja**: Conceptualization; writing—review and editing; resources; funding acquisition; investigation; supervision; data curation.

## CONFLICT OF INTEREST STATEMENT

The authors declare no conflicts of interest.

## Supporting information

Supporting Information S1

Supporting Information S2

## Data Availability

All data supporting the findings of this study are available within the paper and supplementary files published in the Online Repository.
