# Supplementary material for: Type 2 gene expression signature in severe asthma associates with more advanced airway remodeling
Source: Clin Transl Allergy. 2025 Jun 12;15(6):e70060. doi: 10.1002/clt2.70060 (PMC12162258; doi:10.1002/clt2.70060)
Supplement: Supplementary file 2 — Supporting Information S2 [file CLT2-15-e70060-s001.pdf]

Online Supplement

Type 2 gene expression signature in severe asthma associates with more advanced airway remodeling

Bogdan Jakiela, Karolina Górka, Iwona Gross-Sondej, Sławomir Mikrut, Krzysztof Okoń, Piotr Sadowski, Anna Andrychiewicz, Hanna Plutecka, Tomasz Stachura, Grażyna Bochenek, Stanisława Bazan-Socha, Krzysztof Sładek, and Jerzy Soja

# Corresponding authors
Bogdan Jakiela, MD, PhD
Jerzy Soja, MD, PhD
Department of Internal Medicine, Faculty of Medicine,
Jagiellonian University Medical College
Jakubowskiego 2
30-688 Krakow, Poland
Telephone: +48 12 4003054
Fax: +48 12 4003067
e-mail: b.jakiela@uj.edu.pl
e-mail: jerzy.soja@uj.edu.pl

Contents

1. Extended Data Methods .... Page 2
2. Supplementary Tables …....…........................... Page 6
3. Supplementary Figures .... Page 12

1. Extended data Methods

Characteristics of the patients

For this observational cross-sectional study we enrolled 34 patients with severe asthma (median age 50.5 years; females, 56%; all non-smokers), who were managed at the Pulmonology Clinic of the Second Department of Internal Medicine of the Jagiellonian University Medical College, Krakow, Poland. All patients met the criteria for severe asthma according to the Global Initiative for Asthma (GINA) guideline.[1] Chronic obstructive pulmonary disease was excluded. Most patients had adult-onset asthma (79%), with a median duration of the disease 15.5 (8-29.5) years. In all study participants, we performed spirometry at baseline and after short acting β2 agonist (400 µg salbutamol, *inh.*) and measurements of static lung volumes (Jaeger Master Screen; Höchberg, Germany). Basic laboratory parameters, such as blood cell counts or serum IgE, were evaluated using routine methods. Bronchoscopy with endobronchial ultrasound (EBUS) and bronchial sampling was performed in all participants as part of a larger project investigating the usefulness of bronchial thermoplasty in severe asthma.[2] The study protocol was in accordance with the Declaration of Helsinki and its amendments and was approved by the Ethics Committee of the Jagiellonian University Medical College (KBET 122.6120.167.2015). All study participants gave their informed written consent.

Bronchoscopy and airway sample workflow

Fiberoptic bronchoscopies (BF 1T180; Olympus, Tokyo, Japan) were performed according to the guidelines of the American Thoracic Society[3], under local anesthesia (2% lidocaine) and conscious sedation (0.05-0.1 mg of fentanyl and 2.5-5 mg of midazolam, *i.v.*), using the BF-190 fiberscope (Olympus, Tokyo, Japan). In each individual, we performed EBUS, bronchoalveolar lavage, and bronchial biopsy sampling. During the bronchoalveolar lavage procedure, the fiberscope was wedged into the segmental bronchus of the right middle lobe and 0.9% saline solution was instilled (4 portions, 200 mL in total) and suctioned back to collect bronchoalveolar lavage fluid (BALF). Differential cell counts were determined using May-Grünwald Giemsa stained cytospin preparations with counting of ≥1000 cells (Thermo Fisher Scientific, Walthman, MA). BALF supernatant was aliquoted and stored at -80°C for further cytokine measurements. Bronchial biopsies were sampled from the segmental bronchi of the right lower lobe using standard biopsy forceps (EndoJaw; Olympus). The tissue samples were either fixed in 10% buffered formalin for further histology, or mixed with lysis reagent (TRI Reagent, Sigma-Aldrich, St. Louis, MO) and stored at -80°C for further extraction of RNA. In most individuals, we also collected bronchial brushes (Cellebrity Endoscopic Cytology Brushes; Boston Scientific, Marlborough, MA) for the isolation of bronchial epithelial cells. No adverse events were recorded during the bronchoscopy or at follow-up.

Endobronchial ultrasound (EBUS)

EBUS is an advanced bronchoscopic technique that enables detailed visualization of the airway wall and adjacent anatomical structures. The procedure was performed using a 20 MHz radial probe in conjunction with the EU-ME1 ultrasound processor system (Olympus). To ensure the accuracy and consistency of the measurements, EBUS recordings were made in each individual in the four segmental bronchi of the right lower lobe: superior (RB6), anterior basal (RB8), lateral basal (RB9), and posterior basal (RB10). EBUS images were subsequently analyzed using the feature extraction software developed at the University of Science and Technology in Kraków, with further modifications.[4] From the EBUS movie sequences recorded in a given segmental bronchus, five frames with distinctly visible multilayered bronchial wall structures were selected for morphometric analysis. EBUS enables the identification of five layers of the bronchial wall. The inner layers L1 (representing the epithelium and proximal part of the submucosa) and L2 (most of the submucosa, including airway smooth muscle) were analyzed separately, while the outer layers L3, L4, and L5 (L3-5, representing cartilage) were measured together (Fig. 2e in the main text). Layer boundaries were manually delineated by a researcher blinded to the patient's record. The data was converted to millimeter units, and the mean of all measurements was used in the final analysis.

Bronchial biopsy histology

Formalin-fixed tissue samples were embedded in paraffin using standard protocols. Sections of 3 μm thickness were stained with hematoxylin-eosin and immunohistochemically with antibody detecting alpha-smooth muscle actin (α-SMA; M0851; Dako Denmark A/S, Glostrup, Denmark). The specimens were analyzed using Olympus SC 180 camera (Olympus) and the Olympus cellSens Standard 2.3 software. Airway smooth muscle (ASM) measurements were made at 40x magnification, and are expressed as the ratio of α-SMA positive area to the area of the entire section. To ensure reproducibility, morphometric results were averaged from analyzes of four different specimens, with random orientation of cross-sectioned samples to obtain unbiased estimates.[5] Reticular basement membrane thickness was measured as previously described, using imaging system (AnalySIS imaging system; Soft Imaging System GmbH; Germany) and a custom-built application.[4] Images were acquired along the available basement membrane, with a minimum 30 individual measurements performed for each patient sample. Histological examinations were performed by experienced pathologists who were blinded to the clinical data.

RNA isolation and real-time PCR

Total RNA was isolated from samples (bronchial biopsies or *in vitro* grown epithelium) stored in TRI Reagent (Sigma-Aldrich, St. Louis, MO) using the Total RNA Zol-Out Kit (A&A Biotechnology, Gdynia, Poland) and reverse transcribed with the High Capacity cDNA Reverse Transcription Kit (Applied Biosystems, Foster City, CA). Relative mRNA expression was quantified using TaqMan low-density arrays (TLDA; Applied Biosystems; listed in Extended Data Table S1) on the QuantStudio 12K Flex Real-Time PCR System (Applied Biosystems). The qPCR results were processed using ExpressionSuit

Software v1.1 (Thermo Fisher Scientific, Waltham, MA) and normalized to the reference gene *GAPDH*. To simplify, results expressed as 'relative to *GAPDH* (x10^3, $\log_{10}$)' are labeled 'relative expression ($\log_{10}$)' in the graphs. The relative mRNA expression difference between given two groups was calculated using a $2^{-\Delta\Delta CT}$ method, with a 2-fold change (log2fold <-1 or >1; bronchial biopsy data) or a 1.5-fold change (log2fold <-0.585 or >0.585; cell culture experiment data) considered biologically significant. In V-plots, log2fold values were plotted against $-\log_{10}P$-values estimated with the 2-sided t-test (unpaired or paired, as required). Thresholds of biological significance were marked with vertical lines (eg, 2-fold). The results were controlled for multiple comparisons using the Benjamini-Hochberg correction at the false discovery rate threshold (FDRt) q=0.1 (bronchial biopsy data) or q=0.05 (cell culture experiment data). In V-plots, the FDRt threshold was marked with a *red* horizontal line.

Measurements of mediators in BALF

The concentration of the majority of mediators measured in BALF was quantified by Luminex (R&D Systems, Minneapolis, MN). The samples were processed according to the manufacturer's protocols, using Magpix equipment (Luminex Corp., Austin, TX) and xPonent software (Luminex). Standard ELISA assays were used to measure MUC5AC (Cloud-Clone Corp., Katy, TX), Periostin (Biovision Inc., Milpitas, CA), IFN-γ (Thermo Fisher Scientific, Walthman, MA), and TGF-β1 (R&D Systems). The list of measured cytokines, along with the lower limits of quantification, is provided in the Extended Data Table S2. Nearly all measurements of IFN-γ and TNF-α were below the assay threshold and were therefore excluded from the final analysis. To account for sample dilution, data were expressed as pg/mg of protein in BALF, quantified with a NanoDrop spectrophotometer (Thermo Fisher Scientific).

Isolation of human bronchial epithelial cells (HBEC) and in vitro cultures

In cell culture experiments, we used air-liquid interface (ALI) cultures of human bronchial epithelial cells (HBEC) as previously described.[6,7] HBECs were isolated from small superficial bronchial biopsies or bronchial brushes by enzyme digestion (pronase and DNAse; all reagents from Sigma-Aldrich). Cells were expanded in supplemented bronchial epithelial growth medium (BEGM, all media from Lonza, Basel, Switzerland) and cryopreserved. Ultimately, primary HBECs were obtained in ~80% of the patients, but experiments were performed only using 14 cell lines (ie, T2 or nonT2 patients). For the experiment, HBECs were seeded onto transwell membranes (12-well; Costar, Corning Inc., Corning, NY) and initially grown submerged (2 days) in the supplemented BEGM. The cells were then switched to ALI with baso-lateral DMEM/BEGM (1:1) medium supplemented with all-trans-retinoic acid (final 75 nM). Cells were grown for 26 days, with media changes every 2-3 days, resulting in mucociliary differentiation. In the cytokine stimulation model, cells were left untreated (control) or incubated for 4 days with IL-13, IL-17A, or TGF-β1 (1 to 10 pg/mL; all from R&D Systems) added to the basolateral compartment (change of medium after 2 days with fresh cytokines). On day 4, cells were harvested for RNA extraction and further analysis of gene expression (as summarized in Fig. 4a, main text). Here, we checked mRNA expression of 8 important remodeling genes *COL1A1*, *CTGF*, *FGF2*, *FN1*, *MMP9*,

SNAI2, *VIM*, *ZEB*2, and *GAPDH*, using individual TaqMan assays and the QuantStudio platform (Applied Biosystems).

Statistics

Statistical analysis was performed using Statistica 13.1 (TIBCO Software Inc., Palo Alto, CA) and GraphPad Prism 8.4 (GraphPad Software, Inc., La Jolla, CA). Data are presented as medians with quartiles (0.25-0.75) or as numbers with percentages. Results were analyzed using the Kruskall-Wallis (with Dunn's post hoc test) or the Mann-Whitney tests for continuous data, and Fisher's exact test or Chi-squared test for categorical data. mRNA and BALF mediator data were analyzed using the 2-sided t-test (eg, in summary V-plots) or the Mann-Whitney U test, as appropriate. Correlations were typically presented as heat-maps of Pearson (r_P) or Spearmen (r_S) coefficients, with Ward's hierarchical clustering of correlation coefficients. Cell culture data (eg, comparison of cytokine and control condition) were analyzed using a 2-sided paired t-test or RM-ANOVA (with Tukey post hoc test), as indicated. Generally, a *P* value <0.05 was considered significant (more restrictive *P*<0.01 for correlations) using the Benjamini-Hochberg procedure to control for multiple comparisons. For clarity, the number of replicates, statistical methods, and the *P*-value thresholds are indicated in each figure and table legends.

References

1. Global Initiative for Asthma. Global Strategy for Asthma Management and Prevention. Available from: https://ginasthma.org/ [accessed August 16, 2024].
2. Soja J., Górka K., Gross-Sondej I., Jakieła B., Mikrut S., Okoń K., et al. Endobronchial Ultrasound is Useful in the Assessment of Bronchial Wall Changes Related to Bronchial Thermoplasty. *J Asthma Allergy* 2023;**16**:585–95. Doi: 10.2147/JAA.S404254.
3. Guidelines for fiberoptic bronchoscopy in adults. American Thoracic Society. Medical Section of the American Lung Association. *Am Rev Respir Dis* 1987;**136**(4):1066. Doi: 10.1164/ajrccm/136.4.1066.
4. Soja J., Grzanka P., Sładek K., Okoń K., Ćmiel A., Mikoś M., et al. The use of endobronchial ultrasonography in assessment of bronchial wall remodeling in patients with asthma. *Chest* 2009;**136**(3):797–804. Doi: 10.1378/chest.08-2759.
5. Woodruff PG., Innes AL. Quantitative morphology using bronchial biopsies. *European Respiratory Review* 2006:157–61. Doi: 10.1183/09059180.00010106.
6. Jakiela B., Gielicz A., Plutecka H., Hubalewska-Mazgaj M., Mastalerz L., Bochenek G., et al. Th2-type cytokine-induced mucus metaplasia decreases susceptibility of human bronchial epithelium to rhinovirus infection. *Am J Respir Cell Mol Biol* 2014;**51**(2):229–41. Doi: 10.1165/rcmb.2013-0395OC.
7. Jakiela B., Rebane A., Soja J., Bazan-Socha S., Laanesoo A., Plutecka H., et al. Remodeling of bronchial epithelium caused by asthmatic inflammation affects its response to rhinovirus infection. *Sci Rep* 2021;**11**(1):12821. Doi: 10.1038/s41598-021-92252-6.

2. Supplementary Tables

Table S1. TaqMan gene expression assays (Applied Biosystems; Foster City, CA) used in the study.

#	Gene symbol	Assay ID	Gene name
Inflammation panel			
1	*CCR3*	Hs99999027_s1	C-C motif chemokine receptor 3
2	*CLC*	Hs00171342_m1	Charcot-Leyden crystal galectin
3	*CLCA1*	Hs00976287_m1	chloride channel accessory 1
4	*CPA3*	Hs00157019_m1	carboxypeptidase A3
5	*CST1*	Hs00606961_m1	cystatin SN
6	*CXCL8*	Hs00174103_m1	C-X-C motif chemokine ligand 8
7	*DPP4*	Hs00897391_m1	dipeptidyl peptidase 4
8	*ELANE*	Hs00236952_m1	elastase, neutrophil expressed
9	*GAPDH*	Hs99999905_m1	glyceraldehyde-3-phosphate dehydrogenase
10	*GATA2*	Hs00231119_m1	GATA binding protein 2
11	*HDC*	Hs00157914_m1	histidine decarboxylase
12	*IFNG*	Hs00989291_m1	interferon gamma
13	*IL13*	Hs00174379_m1	interleukin 13
14	*IL17A*	Hs00174383_m1	interleukin 17A
15	*IL1B*	Hs01555410_m1	interleukin 1 beta
16	*IL33*	Hs00369211_m1	interleukin 33
17	*IL4*	Hs00174122_m1	interleukin 4
18	*IL6*	Hs00174131_m1	interleukin 6
19	*MPO*	Hs00165162_m1	myeloperoxidase
20	*POSTN*	Hs01566750_m1	periostin
21	*PRSS33*	Hs00541732_m1	protease, serine 33
22	*SERPINB2*	Hs01010736_m1	serpin family B member 2
23	*TNF*	Hs00174128_m1	tumor necrosis factor
24	*TPSAB1*;*TPSB2*	Hs02576518_gH	tryptase beta 2 (gene/pseudogene);tryptase alpha/beta 1
25	*TSLP*	Hs00263639_m1	thymic stromal lymphopoietin
Remodeling panel			
1	*COL1A1*	Hs00164004_m1	collagen type I alpha 1
2	*COL3A1*	Hs00943809_m1	collagen type III alpha 1 chain
3	*CTGF*	Hs00170014_m1	connective tissue growth factor
4	*FGF2*	Hs00266645_m1	fibroblast growth factor 2
5	*FN1*	Hs01549976_m1	fibronectin 1
6	*GDF15*	Hs00171132_m1	growth differentiation factor 15
7	*GDNF*	Hs01931883_s1	glial cell derived neurotrophic factor
8	*MMP2*	Hs01548727_m1	matrix metallopeptidase 2 (gelatinase A, 72kDa gelatinase)
9	*MMP9*	Hs00957562_m1	matrix metallopeptidase 9 (gelatinase B, 92kDa gelatinase)
10	*MUC5AC*	Hs01365616_m1	mucin 5AC, oligomeric mucus/gel-forming
11	*MUC5B*	Hs00861595_m1	mucin 5B, oligomeric mucus/gel-forming
12	*SMAD3*	Hs00969210_m1	SMAD family member 3
13	*SNAI2*	Hs00161904_m1	snail family transcriptional repressor 2
14	*TGFB1*	Hs00998133_m1	transforming growth factor beta 1
15	*TIMP1*	Hs01092512_g1	TIMP metallopeptidase inhibitor 1
16	*VEGFA*	Hs00900055_m1	vascular endothelial growth factor A
17	*VIM*	Hs00958111_m1	vimentin
18	*ZEB2*	Hs00207691_m1	zinc finger E-box binding homeobox 2

Table S2. List of bronchoalveolar lavage fluid (BALF) mediators analyzed in the study.

#	Short name	Full/formal name	Method	LLQ (pg/mL)	Included in the analysis
1	CD146	Cell surface glycoprotein MUC18	Luminex	148,17	Yes
2	CHI3L1	Chitinase-3-like protein 1	Luminex	392,32	Yes
3	CXCL8	Interleukin-8	Luminex	4,19	Yes
4	ECP	Eosinophil cationic protein	ELISA	1,56	Yes
5	FGF-2	Fibroblast growth factor 2	Luminex	2,56	Yes
6	IFN-γ	Interferon gamma	ELISA	0,06	No, all samples negative
7	IL-13	Interleukin-13	Luminex	218,98	Yes
8	IL-4	Interleukin-4	Luminex	12,29	Yes
9	IL-5	Interleukin-5	Luminex	3,49	Yes
10	IL-6	Interleukin-6	Luminex	4,54	Yes
11	MMP-2	Matrix metalloproteinase-2	Luminex	257,49	Yes
12	MMP9	Matrix metalloproteinase-9	Luminex	75,53	Yes
13	MPO	Myeloperoxidase	Luminex	81,17	Yes
14	MUC5AC	Mucin-5AC	ELISA	15	Yes
15	NGAL	Neutrophil gelatinase-associated lipocalin	Luminex	90,64	Yes
16	Periostin	Periostin	ELISA	78	Yes
17	TGF-β1	Transforming growth factor beta-1	ELISA	4,61	Yes
18	TIMP-1	Metalloproteinase inhibitor 1	Luminex	14,89	Yes
19	TNF-α	Tumor necrosis factor	Luminex	6,29	No, 90% negative

Table S2 footnote: Abbreviations: LLQ, lower limit of quantification. All luminex assays were provided by Bio-Techne (Minneapolis, MN; R&D Systems Luminex assays). ELISA sets were purchased in Cloud-Clone Corp. (Katy, TX; MUC5AC), Thermo Fisher Scientific (Waltham, MA; Invitrogen: IFN-γ), Bio-Techne (R&D Systems: TGF-β1), MyBioSource (San Diego, CA; ECP), and Biovision Inc. (Milpitas, CA; Periostin).

Table S3. Clinical characteristics and bronchoalveolar lavage fluid (BALF) cell differential in asthma patient stratified based on T2 and T3 gene expression signature in the bronchial biopsy.

	**T2-high** (n=13)	**T3-high** (n=5)	**Low-Inflammatory** (n=10)	*P*-value	Test	*P*-value **T2 v T3**	*P*-value **T2 v Low**	*P*-value **T3 v Low**	Test
Age (years)	51 [43-54.5]	45 [29.5-56.5]	48.5 [41-61]	0.7626	K-W	0,7028	0,7381	0,4396	M-W
Females (n, %)	7 (53.8%)	2 (40%)	7 (70%)	0.5136	Chi2	>0.9999	0.6693	0.3287	Fisher
BMI (kg/m^2)	26.1 [23.5-29.0]	20.2 [19.4-23.3]	27.4 [23.9-31.9]	**0.0254**	K-W	**0,0044**	0,7381	**0,0280**	M-W
Age of onset (year)	35 [12-43]	25 [19.5-27.5]	34 [9-50]	0.6854	K-W	0,4430	0,5629	0,5941	M-W
Asthma duration (years)	15 [8-34]	23 [8.5-29]	16 [4-36]	0.9695	K-W	0,9241	0,9758	0,7679	M-W
Positive SPT (n, %)	8 (61.5%)	2 (40%)	5 (50%)	0.6862	Chi2	0.6078	0.6850	>0.9999	Fisher
ACT (score)	12 [8-16]	13 [11.5-18]	13.5 [9-18.3]	0.7139	K-W	0,6235	0,6049	0,9451	M-W
AQLQ (score)	3.31 [2.63-4.92]	4.69 [3.72-6.29]	4.14 [3.19-4.90]	0.3353	K-W	0,2017	0,5629	0,3037	M-W
Number of exacerbations (n/year)	4 [3-8]	4 [1-8]	5 [3-8]	0.8624	K-W	0,6331	0,9758	0,6787	M-W
ICS (µg/d)[1]	1800 [1000-2300]	1600 [1060-2250]	1920 [1300-2550]	0.7817	K-W	1,0000	0,5629	0,5941	M-W
OCS (mg/d)[2]	4 [0-14]	8 [0-20]	4 [0-20]	0.9483	K-W	0,7750	0,8793	0,8591	M-W
OCS (n, %)	9 (69.2%)	3 (60%)	5 (50%)	0.6448	Chi2	>0.9999	0.4173	>0.9999	Fisher
LABA (n, %)	13 (100%)	5 (100%)	10 (100%)	n.a.	Chi2	>0.9999	>0.9999	>0.9999	Fisher
LAMA (n, %)	6 (46.2%)	2 (40%)	4 (40%)	0.9476	Chi2	>0.9999	>0.9999	>0.9999	Fisher
SAMA (n, %)	4 (30.8%)	1 (20%)	1 (10%)	0.4830	Chi2	>0.9999	0.3394	>0.9999	Fisher
LTRA (n, %)	4 (30.8%)	0 (0%)	3 (30%)	0.3623	Chi2	0.2778	>0.9999	0.5055	Fisher
Blood eosinophils (cells/µL)	383 [153-467]	98 [18-114]	139 [41-202]	**0.0069**	K-W	**0,0068**	**0,0178**	0,2065	M-W
Blood basophils (cells/µL)	30 [18-65]	37 [13-47]	23 [16-31]	0.4353	K-W	0,5663	0,2569	0,5135	M-W
Blood neutrophils (cells/µL)	4652 [3954-5749]	7720 [7321-11555]	4183 [2381-6840]	**0.0058**	K-W	**0,0002**	0,8315	**0,0080**	M-W
Serum total IgE (IU/mL)	85.5 [38.6-163]	17.8 [17.8-167]	145 [30.9-556]	0.1797	K-W	0,1037	0,6744	0,1292	M-W
Serum CRP (mg/L)	1.3 [1.1-2.3]	12.5 [2.7-35.7]	3.7 [1.0-4.9]	**0.0370**	K-W	**0,0068**	0,5123	0,1119	M-W
BALF analysis									
BALF cell count (10^3 cells/mL)	116 [92-188]	152 [112-310]	166 [127-268]	0.2832	K-W	0,5028	0,1151	0,6787	M-W
Macrophages (% of non epi. cells)	93.1 [86.6-96.6]	78.2 [47.3-90.4]	95.8 [82.7-97.1]	0.0503	K-W	**0,0264**	0,4833	**0,0400**	M-W
Neutrophils (% of non epi. cells)	0.52 [0.36-1.87]	8.4 [5.16-31.1]	1.11 [1.0-1.69]	0.0014	K-W	**0,0002**	0,1306	**0,0013**	M-W
Eosinophils (% of non epi. cells)	1.53 [0.10-2.99]	0.30 [0.14-0.41]	0.15 [0.1-0.28]	0.1711	K-W	0,3359	0,1010	0,2544	M-W

Lymphocytes (% of non epi. cells)	4.19 [0.98-7.18]	14.1 [3.82-21.2]	3.26 [1.93-14.1]	0.2060	K-W	0,0754	0,5629	0,3097	M-W
BALF Eosinophils >2% (n, %)	5 (38.5%)	0 (0%)	1 (10%)	0.1119	Chi2	0,2489	0,1790	>0.9999	Fisher
BALF Neutrophils >3% (n, %)	0 (0%)	5 (100%)	1 (10%)	**<0.0001**	Chi2	**0.0001**	0.4348	**0.0020**	Fisher
Airway inflammation, E/N/P (n)[3]	5/0/8	0/5/0	1/1/8	**<0.0001**	Chi2	**0.0001**	0.1890	**0.0036**	Chi2

Table S3 footnotes: Abbreviations: ACT, Asthma Control Test; AQLQ, Asthma Quality of Life Questionnaire; BALF, bronchoalveolar lavage fluid; BMI, body mass index; CRP, C-reactive protein; ICS, inhaled corticosteroids; LABA, Long-acting β adrenoceptor agonists; LAMA, long-acting muscarinic antagonists; LTRA, leukotriene receptor antagonists; n.a., non applicable; OCS, oral corticosteroids; SAMA, Short-acting muscarinic-antagonists; SPT, skin prick tests.
References: 1, dose adjusted for fluticasone; 2, dose adjusted for methylprednisolone; 3, incidence of lower airway inflammatory phenotypes in asthma patients based of BALF cell analysis: E, eosinophilic (>2% eosinophils, ≤3% neutrophils); N, neutrophilic (≤2% eosinophils, >3% neutrophils); P, pauci-granulocytic (≤2% eosinophils, ≤3% neutrophils).
Statistics: Data are presented as medians [Q1-Q3 quartiles] or numbers (n, percentage). Kruskal-Wallis (K-W) and Mann-Whitney (M-W) tests were used to compare continuous data. Contingency table statistics was assessed with Chi-square (Chi2) and Fisher's exact test. Significant differences (*P*<0.05) are highlighted with a red font. Patients with T2/T3 signature were excluded from analysis due to low sample size (n=2).

Table S4. Lung function and airway structural measures in asthma patients with different immune endotypes.

	**T2-high** (n=13)	**T3-high** (n=5)	**Low-Inflammatory** (n=10)	*P*-value	Test	*P*-value **T2 v T3**	*P*-value **T2 v Low**	*P*-value **T3 v Low**	Test
Lung function									
FVC (% predicted)	74.0 [66.2-84.2]	79.0 [62.9-103.5]	89.9 [82.8-98.3]	0.1275	K-W	0.7028	**0.0422**	0.3710	M-W
FEV1 (% predicted)	59.0 [46.6-72.5]	65.0 [41.7-95.8]	73.9 [59.5-92.6]	0.1629	K-W	0,5028	**0,0493**	0,6787	M-W
FEV1%FVC before BD	62.6 [53.6-72.8]	63.8 [54.3-82.0]	69.7 [59.7-75.2]	0.5835	K-W	0,7028	0,3128	0,8591	M-W
FEV1%FVC after BD	74.7 [56.8-79.2]	65.0 [57.7-82.4]	71.9 [63.9-74.3]	0.8698	K-W	0,8490	0,7844	0,5135	M-W
FAO (n, %)	6 (46.2%)	3 (60%)	3 (30%)	0.5136	Chi2	>0.9999	0.6693	0.3287	Fisher
FEV1.rev (% change after BD)	9.3 [1.5-17.3]	1.4 [-5.3-10.7]	4.4 [1.9-12.1]	0.3348	K-W	0,1734	0,5224	0,3097	M-W
TLC (% predicted)	97.0 [89.2-112.5]	96.0 [89.2-118.3]	115.2 [107.1-124.5]	**0.0388**	K-W	0,9241	**0,0121**	0,1292	M-W
RV (% predicted)	119.0 [95.0-181.9]	135.3 [130.3-154.4]	144.6 [122.8-193.5]	0.2059	K-W	0,3359	0,0883	0,9530	M-W
RV/TLC (ratio)	0.428 [0.343-0.468]	0.422 [0.379-0.500]	0.456 [0.369-0.5	0.7512	K-W	0,7750	0,5224	0,6787	M-W
EBUS measurements									
L1 (mm)	0.182 [0.179-0.185]	0.185 [0.173-0.191]	0.175 [0.170-0.183]	0.1488	K-W	0,3873	0,1151	0,1645	M-W
L2 (mm)	0.203 [0.198-0.211]	0.204 [0.194-0.215]	0.186 [0.179-0.201]	**0.0346**	K-W	0,9241	**0,0178**	0,0553	M-W
L3-5 (mm)	0.945 [0.880-1.009]	0.969 [0.897-1.045]	0.898 [0.831-0.953]	0.1376	K-W	0,5663	0,1010	0,1292	M-W
Bronchial biopsy histology									
ASM (% biopsy cross-sect. area)	10.60 [6.42-17.34]	7.09 [2.69-11.33]	5.78 [3.86-9.31]	0.0508	K-W	0,1734	**0,0178**	0,8591	M-W
RBM thickness (μm)	6.89 [6.67-7.89]	7.06 [5.45-7.66]	6.38 [5.94-6.84]	0.0776	K-W	0,5663	**0,0178**	0,5135	M-W

Table S4 footnotes: Abbreviations: ASM, airway smooth muscle; BD, bronchodilator (short acting β2-agonist); FAO, fixed airway obstruction (defined as FEV1%FVC post BD <70%); FEV1, forced expiratory volume in 1 second; FEV1.rev, reversibility of FEV1; FVC, forced vital capacity; RBM, reticular basement membrane; RV, residual volume; TLC, total lung capacity.
Statistics: Data are presented as medians [Q1-Q3 quartiles] or numbers (n, percentage). Kruskal-Wallis (K-W) and Mann-Whitney (M-W) tests were used to compare continuous data. Contingency table statistics (only for FAO) was assessed with Chi-square (Chi2) and Fisher's exact test. Significant differences (P<0.05) are highlighted with a red font. Patients with T2/T3 signature not were no t analyzed due to low sample size (n=2).

Table S5. Important clinical an airway structural characteristics in asthma patient clusters identified based on baseline (ie, without addition of cytokines) expression of remodeling genes in ALI-cultured HBECs (as in the main text Fig. 4f).

	**CL1** (higher expression of remodeling genes in cultured HBECs, n=7)	**CL2** (lower expression of remodeling genes in cultured HBECs, n=7)	Median difference or odds ratio (95% CI)	*P*-value
Age (years)	45 [41-52]	55 [44-60]	-10 (-15, 7)	0.2424
Females (n, %)	5 (71.4%)	3 (42.9%)	3.33 (0.31, 24.1)	0.5921
BMI (kg/m^2)	25.1 [23.9-29.3]	28.7 [22.1-34.6]	-3.6 (-10.1, 5.1)	0.7092
Asthma duration (years)	15 [5-20]	16 [2-45]	-1 (-30, 13)	0.7261
Positive SPT (n, %)	3 (42.9%)	5 (71.4%)	0.3 (0.04, 3.2)	0.5921
Number of exacerbations (n/year)	4 [3-8]	5 [2-9]	-1 (-5, 5)	>0.9999
ICS (μg/d)[1]	2000 [1000-2200]	2400 [1800-3400]	-400 (-1400, 600)	0.2984
OCS (mg/d)[2]	8 [0-16]	0 [0-16]	8 (-12, 16)	0.3351
Blood eosinophils (cells/μL)	328 [97-452]	173 [154-208]	155 (-111, 298)	0.3176
Serum total IgE (IU/mL)	70.9 [35.3-254]	64.1 [40.6-549]	6.8 (-513, 197)	0.8368
Serum CRP (mg/L)	1.05 [1.0-3.95]	1.90 [1.0-10.1]	-0.85 (-9.1, 2.6)	0.2949
BALF analysis				
BALF cell count (10^3 cells/mL)	136 [92-240]	172 [96-188]	-36 (-104, 80)	0.5117
Macrophages (% of non epi. cells)	93.9 [82.8-96.5]	96.4 [92.5-97.1]	-2.5 (-13.9, 3.4)	0.3829
Neutrophils (% of non epi. cells)	1.1 [0.5-1.3]	1.4 [0.9-1.9]	-0.3 (-1.2, 0.2)	0.2086
Eosinophils (% of non epi. cells)	0.2 [0.1-2.1]	0.2 [0.1-1.5]	0 (-1.3, 2.1)	0.8916
Lymphocytes (% of non epi. cells)	5.0 [2.7-13.0]	2.0 [1.1-5.5]	3.0 (-2.5, 11.6)	0.1964
Lung function				
FEV1%FVC before BD	65.1 [52.0-74.3]	62.6 [59.0-70.4]	2.5 (-16, 15.3)	0.9015
FEV1%FVC after BD	70.8 [53.9-80.8]	66.2 [62.0-74.9]	4.6 (-12.7, 13.1)	>0.9999
FAO (n, %)	3 (42.9%)	4 (57.1%)	0.56 (0.09, 5.92)	>0.9999
FEV1 (% predicted)	62.0 [54.8-93.0]	70.0 [59.0-75.0]	-8 (-17.8, 23)	0.8747
FEV1.rev (% change after BD)	4 [-1-21]	5 [1-18.6]	-1 (-13.8, 12)	0.9015
Airway structure				
L1 (mm)	0.177 [0.169-0.182]	0.177 [0.174-0.187]	0 (-0.013, 0.006)	0.3829
L2 (mm)	0.184 [0.179-0.200]	0.206 [0.201-0.213]	-0.022 (-0.03, 0.004)	0.0973
L3-5 (mm)	0.866 [0.824-0.945]	0.956 [0.867-1.007]	-0.089 (-0.174, 0.03)	0.1649
ASM (% biopsy cross-sect. area)	6.34 [4.14-9.56]	4.97 [3.67-10.66]	1.37 (-6.52, 5.08)	0.9015
RBM thickness (μm)	6.00 [5.95-7.83]	6.69 [6.02-7.65]	-0.69 (-1.68, 1.14)	0.2593

Table S5 footnotes: Abbreviations: ALI, air-liquid interface; ASM, airway smooth muscle; BALF, bronchoalveolar lavage fluid; BD, bronchodilator (short acting β2-agonist); BMI, body mass index; CRP, C-reactive protein; FAO, fixed airway obstruction (defined as FEV1%FVC post BD <70%); FEV1, forced expiratory volume in 1 second; FEV1.rev, reversibility of FEV1; FVC, forced vital capacity; ICS, inhaled corticosteroids; OCS, oral corticosteroids; SPT, skin prick test. RBM, reticular basement membrane;
References: 1, dose adjusted for fluticasone; 2, dose adjusted for methylprednisolone.
Statistics: Data are presented as medians [Q1-Q3 quartiles] or numbers (n, percentage). Median differences with Mann-Whitney statistics (for continuous data) or odds ratios with contingency table statistics (for categorical data; Fisher's exact test) are presented in separate columns. No significant differences (P<0.05) were recorded.

3. Supplementary Figures

Figure S1

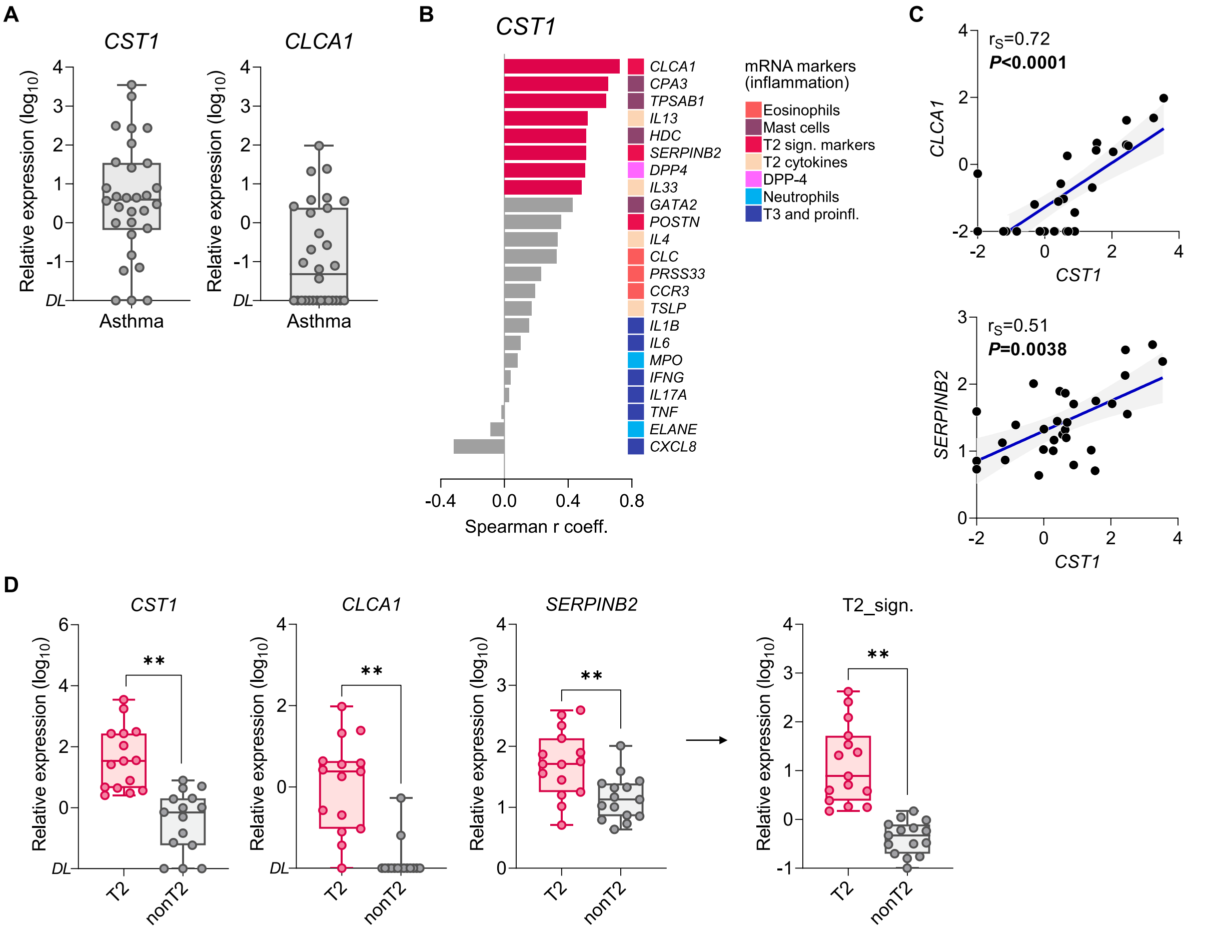

Figure S1 legend. **Expression of T2 genes in bronchial biopsies of severe asthma patients and T2-signature score**. (**A**) Bronchial biopsy mRNA expression of *CST1* and *CLCA1* in all patients studied (n=30). Data are presented as median with quartiles. DL, detection limit. (**B**) Bar chart of pairwise correlations between mRNA expression of *CST1* and other inflammatory genes studied. Significant (P<0.01) correlations are marked in *red*. These include other T2-markers *CLCA1* and *SERPINB2* (but not *POSTN*), key mast cell markers (eg, *CPA3*), *DPP4*, *IL13*, and *IL33*. (**C**) *CLCA1* and *SERPINB2* were the top T2-genes significantly positively correlated with *CST1*. r_S, Spearmen coefficient. n=30, fitted regression line with 95% CI range. (**D**) T2-signature score (T2_sign.) was calculated as the average expression of the top correlated T2 genes: *CST1*, *CLCA1*, and *SERPINB2*. Patients were then stratified as having T2 or nonT2 signature based on the median value of this score (see main text for details). Here, we show mRNA expression of *CST1*, *CLCA1*, *SERPINB2*, and T2-sign. in the two asthma groups. **P<0.01 (Mann-Whitney).

Figure S2

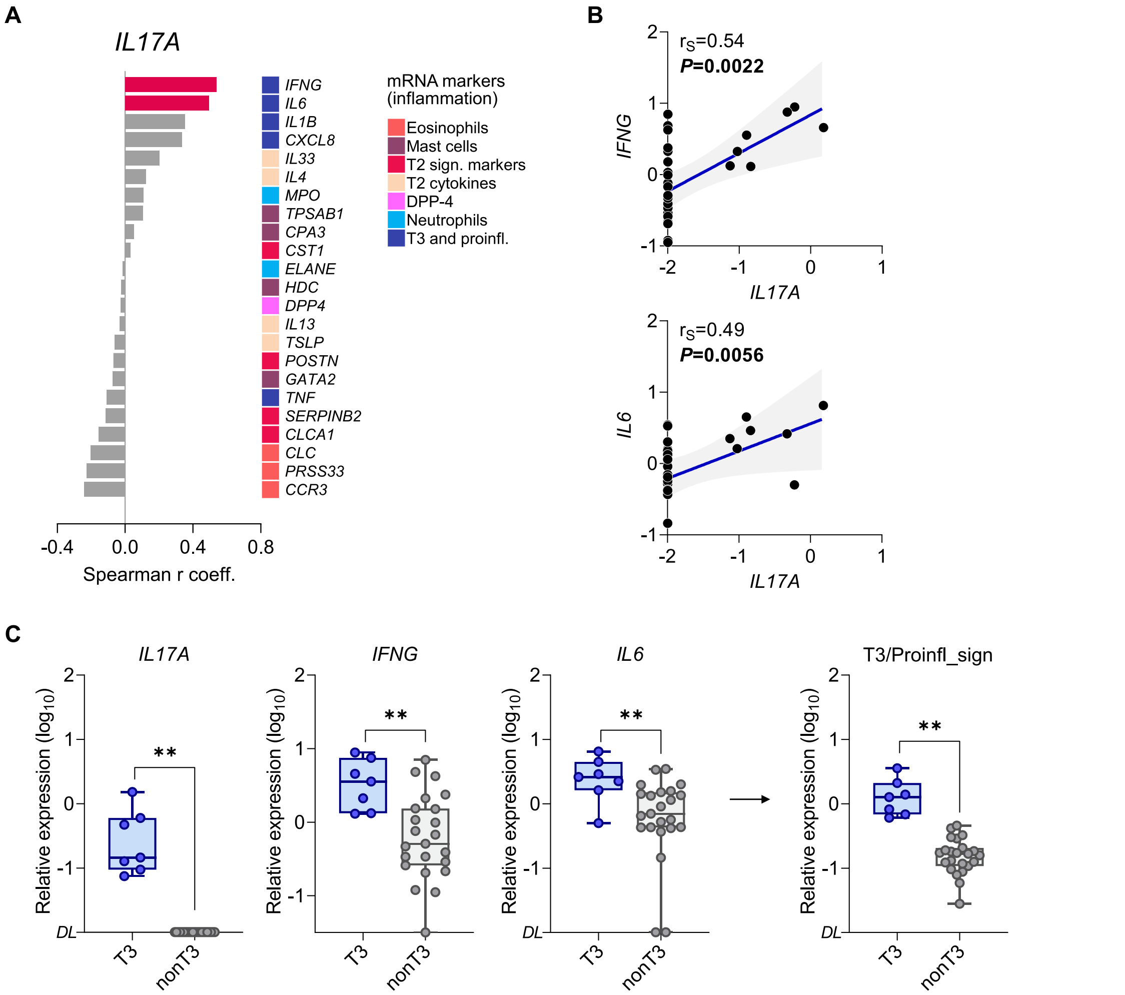

Figure S2 legend. **Expression of *IL17A* and proinflammatory genes in bronchial biopsies and T3/proinfl-signature score.** (**A**) Bar chart of pairwise correlations between mRNA expression of *IL17A* and other inflammatory genes. Significant (P<0.01) correlations are marked in *red*. These include only *IFNG* and *IL6*. (**B**) Scatter plots showing correlation of *IL17A* with *IFNG* and *IL6*. r_S, Spearmen coefficient. n=30, fitted regression line with 95% CI range. (**C**) T3/Proinflammatory-signature score (T3/Proinfl._sign.) was calculated as the average expression of *IL17A*, *IFNG*, and *IL6*. Only 7 patients had detectable *IL17A* mRNA in the bronchial biopsy and were thus classified as having T3 asthma. These patients were characterized by significantly increased expression of *IFNG* and *IL6*, and as a consequence also markedly elevated T3/Proinfl. signature score. Here, we summarize mRNA expression of *IL17A*, *IFNG*, *IL6*, and T3/Proinfl.-sign. in patients with T3 and nonT3 asthma. ∗∗P<0.01 (Mann-Whitney).

Figure S3

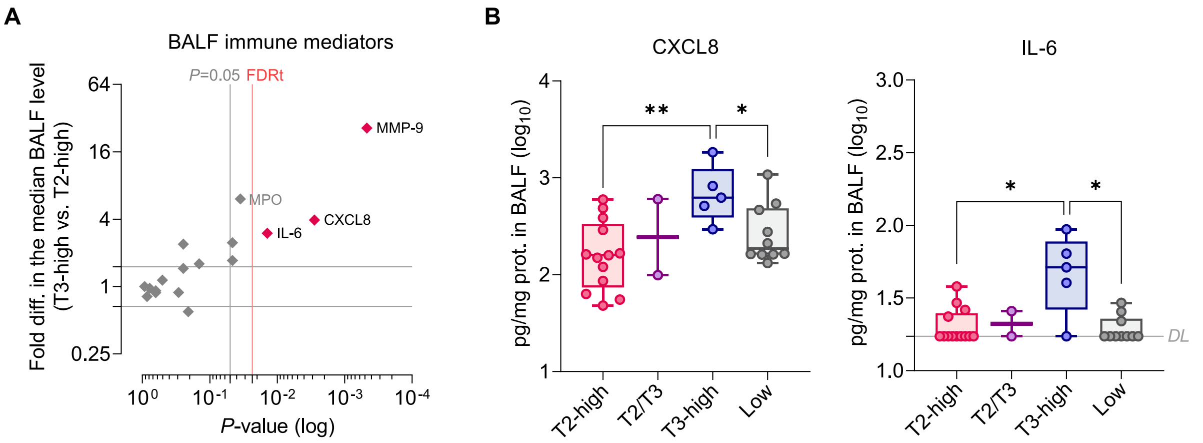

Figure S3 legend. **Increased BALF levels of proinflammatory cytokines in T3-high asthma.** (**A**) Fold difference in the bronchoalveolar lavage fluid (BALF) concentration of immune mediators in T3-high asthma (n=5) compared to T2-high (n=13). Vertical lines indicate significance thresholds (Mann-Whitney, *P*=0.05; FDRt, false discovery rate threshold at *q*=0.01). Horizontal lines mark 1.5-fold difference. (**B**) BALF concentration of CXCL8 (IL-8) and IL-6 in asthma patients with different immune endotypes based on bronchial biopsy gen expression. T2/T3 not included in analysis due to low sample size. Mann-Whitney: ∗∗*P*<0.01, ∗*P*<0.05. DL, detection limit.

Figure S4

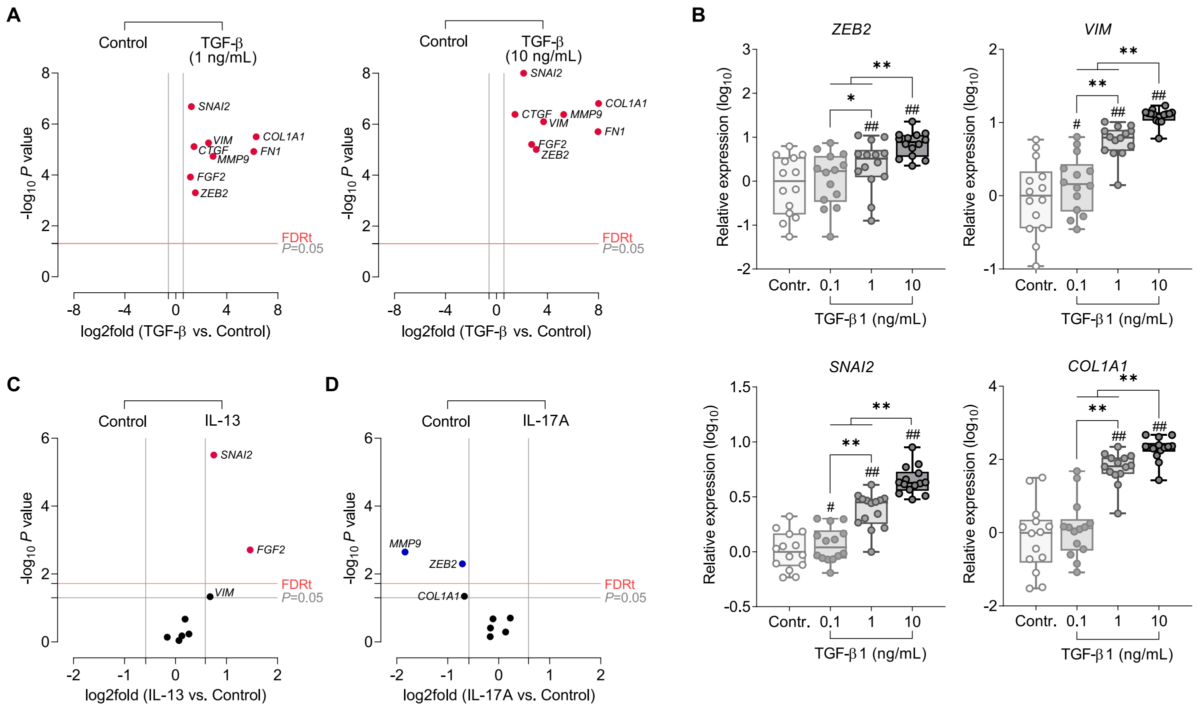

Figure S4 legend. **Changes in mRNA expression of remodeling genes in cytokine-stimulated bronchial epithelial cells (HBEC)**. (**A**) Relative expression difference in HBECs incubated (4 days) with TGF-β1 (1 or 10 ng/mL) compared to untreated control. All studied genes were significantly induced by TGF-β1 (*red*). (**B**) mRNA expression (relative to control) of *ZEB2*, *VIM*, *SNAI2*, and *COL1A1* in control or TGF-β1 treated cells. Data shown as medians and quartiles (n=14): RM-ANOVA (Tukey): #P<0.05, ##P<0.01 compared to control. ∗P<0.05, ∗∗P<0.01 as assigned. (**C**) *SNAI2* and *FGF2* mRNA was significantly induced in HBECs exposed to IL-13. In contrast (**D**), IL-17A treatment resulted in decreased expression of *ZEB2* and *MMP9*. Statistics in ‘a’, “c’ and ‘d”: n=14, 2-sided paired t-test, FDRt q=0.05. Vertical lines indicate 1.5-fold difference.

Figure S5

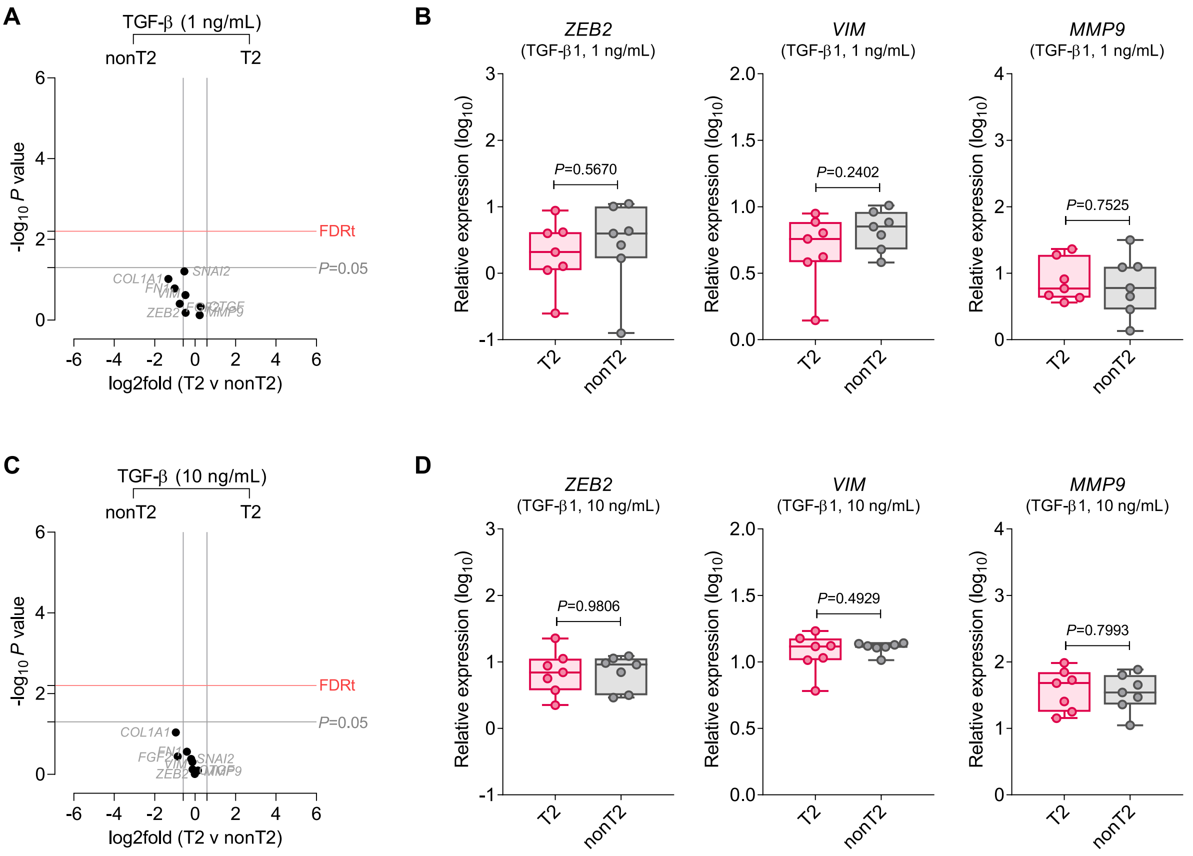

Figure S5 legend. **Similar TGF-β1 response in bronchial epithelial cells from severe asthma patients with T2 and nonT2 immune signature**. (**A**) V-plot showing no difference in mRNA expression of the studied remodeling genes in HBECs (treated with TGF-β1, 1 ng/mL) from T2 patients (n=7) compared to nonT2 (n=7). 2-sided t-test, FDRt *q*=0.05. Vertical lines indicate 1.5-fold difference. (**B**) Individual mRNA expression data for *ZEB2*, *VIM*, and *MMP9* in TGF-β1 (1 ng/mL) stimulated samples. Data presented as medians and quartiles. 2-sided t-test. (**C**, **D**) Similar to 'a' and 'b' if stimulated with 10 ng/mL TGF-β1.

Figure S6

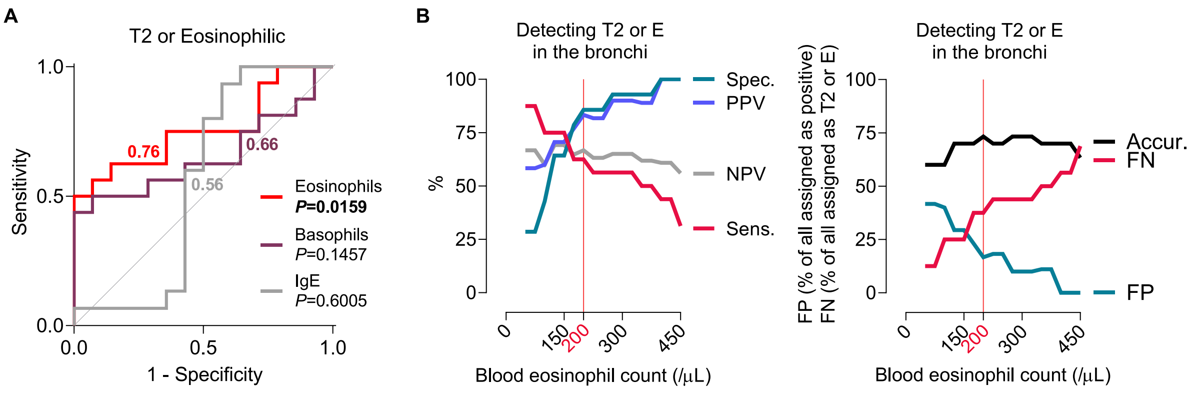

Figure S6 legend. **Usefulness of surrogate biomarkers for the detection of T2 and/or eosinophilic asthma**. (**A**) Receiver operating characteristic (ROC) curves of blood eosinophil and basophil counts, and serum IgE to predict patients with T2 signature in the bronchial brush or BALF eosinophilia (E, eosinophilic). The numbers represent the area under the ROC curve values. (**B**) Diagnostic test performance of blood eosinophil counts in detection of T2 signature in bronchial biopsy and/or BALF eosinophilia. Sens., sensitivity; Spec., specificity; PPV, positive predictive value; NPV, negative predictive value; Accur., accuracy; FN, false negative; FP, false positive. Cutoff 150 cells/μL: Sens., 75.0%; Spec., 64.3%; PPV, 70.6%; NPV, 69.2%; FN, 25.0%; FP, 29.4%; Accur., 70.0%. Cutoff 200 cells/μL: Sens., 62.5%; Spec., 85.7%; PPV, 83.3%; NPV, 66.7%; FN, 37.5%; FP, 16.7%; Accur., 73.3%.
